# Harmonization of Protocols for Multi-Species Organoid Platforms to Study the Intestinal Biology of *Toxoplasma gondii* and Other Protozoan Infections

**DOI:** 10.3389/fcimb.2020.610368

**Published:** 2021-02-22

**Authors:** David Holthaus, Estefanía Delgado-Betancourt, Toni Aebischer, Frank Seeber, Christian Klotz

**Affiliations:** FG 16: Mycotic and Parasitic Agents and Mycobacteria, Robert Koch-Institute, Berlin, Germany

**Keywords:** intestinal epithelium, host-pathogen interactions, epithelial barrier function, intestinal organoid, protozoa, *Toxoplasma gondii*, *Giardia* spp.

## Abstract

The small intestinal epithelium is the primary route of infection for many protozoan parasites. Understanding the mechanisms of infection, however, has been hindered due to the lack of appropriate models that recapitulate the complexity of the intestinal epithelium. Here, we describe an *in vitro* platform using stem cell-derived intestinal organoids established for four species that are important hosts of Apicomplexa and other protozoa in a zoonotic context: human, mouse, pig and chicken. The focus was set to create organoid-derived monolayers (ODMs) using the transwell system amenable for infection studies, and we provide straightforward guidelines for their generation and differentiation from organ-derived intestinal crypts. To this end, we reduced medium variations to an absolute minimum, allowing generation and differentiation of three-dimensional organoids for all four species and the subsequent generation of ODMs. Quantitative RT-PCR, immunolabeling with antibodies against marker proteins as well as transepithelial-electrical resistance (TEER) measurements were used to characterize ODM’s integrity and functional state. These experiments show an overall uniform generation of monolayers suitable for *Toxoplasma gondii* infection, although robustness in terms of generation of stable TEER levels and cell differentiation status varies from species to species. Murine duodenal ODMs were then infected with *T. gondii* and/or *Giardia duodenalis*, two parasites that temporarily co-inhabit the intestinal niche but have not been studied previously in cellular co-infection models. *T. gondii* alone did not alter TEER values, integrity and transcriptional abundance of tight junction components. In contrast, in *G. duodenalis*-infected ODMs all these parameters were altered and *T. gondii* had no apparent influence on the *G. duodenalis*-triggered phenotype. In conclusion, we provide robust protocols for the generation, differentiation and characterization of intestinal organoids and ODMs from four species. We show their applications for comparative studies on parasite-host interactions during the early phase of a *T. gondii* infection but also its use for co-infections with other relevant intestinal protozoans.

## Introduction

*Toxoplasma gondii* and *Giardia duodenalis* are two of the most common parasites associated with protozoan disease in humans and animals (Taylor and Webster, 1998; Dubey, 2010; Cacciò and Sprong, 2011; Geurden and Olson, 2011; Torgerson and Macpherson, 2011; Kirk et al., 2015; Torgerson et al., 2015; Cacciò et al., 2018). Both parasites are zoonotic pathogens and share the same route of infection by oral uptake, and both organisms initiate infection in the small intestine, thus temporarily sharing the same habitat. However, reports on the impact of potential co-infections are scarce and have been limited by difficulties in translation of animal experiments or adequate *in vitro* model systems. In general, despite the impact and frequency of these diseases, research mostly relies on animal models or cancer cell lines that mostly do not recapitulate the situation of naturally occurring infections (Klotz et al., 2012; Delgado Betancourt et al., 2019).

The intestinal epithelium is characterized by a villus-crypt axis that is in constant cell renewal, with cells migrating from the crypt to the tip of the villi while differentiating. Beside stem cells, five major cell types can be distinguished: the absorptive enterocyte and cells of the secretory lineage such as Paneth, goblet, enteroendocrine, and Tuft cells. Mimicking this complex environment is key in understanding intestinal homeostasis and disease (Clevers, 2016). Three-dimensional (3D) organoids are *in vitro-*generated stem cell-based multicellular models that replicate the organ-specific architecture and functionality (Clevers, 2016). They are *in vitro* systems that promise to improve the reliability of host-pathogen models and consequently are currently used to study host interactions with pathogens, including protozoa (Dutta and Clevers, 2017; Hill and Spence, 2017; Heo et al., 2018; Luu et al., 2019; Martorelli Di Genova et al., 2019; Wilke et al., 2019; Kraft et al., 2020). Embedded in an extracellular matrix and supplemented with growth factors, organoids allow almost indefinite propagation of healthy and diseased primary tissues of various hosts (Sato et al., 2011a; VanDussen et al., 2015; Derricott et al., 2019).

For infection of 3D organoids various techniques have been reported (Hill et al., 2017; Williamson et al., 2018; Co et al., 2019; Luu et al., 2019). All of them have shortcomings that can limit translational success and relevance, including the difficulty of accessing the lumen, decreased viability and lower infection yields. To address some of these drawbacks, two-dimensional organoid-derived monolayers (ODMs) have been developed (Moon et al., 2014; VanDussen et al., 2015). By seeding fragmented organoids or single cells on top of pre-coated transwell filters, it is possible to generate compartmentalized infection models that possess most of the advantages of 3D organoid models while also being easily up-scalable and accessible. The use of ODMs has already enabled elementary breakthroughs in apicomplexan research such as *in vitro* sexual reproduction of *Cryptosporidium* sp. (Heo et al., 2018; Wilke et al., 2019).

*T. gondii* is capable of infecting virtually any nucleated cell of vertebrate hosts, including humans, rodents, birds and livestock (Lindsay and Dubey, 2007). Approximately 30% of the human population is infected with the parasite (Montoya and Liesenfeld, 2004), and more than 50% of *T. gondii* infections are associated with the consumption of contaminated meat products from wildlife and livestock (Dubey and Jones, 2008; Kijlstra and Jongert, 2008; Stelzer et al., 2019). The life cycle is complex and encompasses an asexual cycle of fast replicating tachyzoite and dormant tissue cysts comprising of bradyzoites, and sexual development in the feline definite host producing dormant oocysts comprising infectious sporozoites. After ingestion, bradyzoites and sporozoites encounter the intestinal epithelium where they disseminate to different tissues throughout the body (Delgado Betancourt et al., 2019). Frequency of gastrointestinal symptoms is unclear as they may not be recognized, but if so, appear unspecific including abdominal pain and diarrhea (Glover et al., 2017). However, in animals, in particular in laboratory mice, gastrointestinal immunopathology caused by severe inflammation is frequently observed (Schreiner and Liesenfeld, 2009). The exact interaction of the parasite with the intestinal epithelial layer is still unclear (Barragan et al., 2005; Lambert and Barragan, 2010; Gregg et al., 2013), although the parasite is reported to exploit several mechanisms to penetrate the epithelium, such as transepithelial migration (Barragan and Sibley, 2002; Barragan et al., 2005) and modulation of junctional proteins (Weight and Carding, 2012; Weight et al., 2015; Briceño et al., 2016). However, most of these observations have been performed using immortalized cell lines, which in many cases lack the architecture and properties of the cell populations found in the intestine (Pageot et al., 2000; Balimane and Chong, 2005; Hill and Spence, 2017). We and others have therefore proposed the use of intestinal organoids as potentially more relevant cellular systems to study these early events of infection (Klotz et al., 2012; Delgado Betancourt et al., 2019; Luu et al., 2019).

*G. duodenalis* is a species complex with zoonotic potential that infects multiple species of mammals including humans (Cacciò et al., 2018). Parasite prevalence depends largely on hygiene standards but may reach very high numbers of more than 50% in the studied animal or human population (Cacciò and Sprong, 2011; Geurden and Olson, 2011; Helmy et al., 2018). It is therefore likely that initial infection with *T. gondii* frequently coincides with *G. duodenalis* infection. Moreover, recent evidence of co-occurrence of environmental stages of *G. duodenalis* and *T. gondii* in water samples (Pineda et al., 2020) suggests the possibility of simultaneous co-infection of hosts with these two parasites. After ingestion of *G. duodenalis* cysts, trophozoites excyst and are released in the intestinal lumen where the infection is established. *G. duodenalis* trophozoites colonize and replicate in close proximity to the intestinal epithelium. Yet, in contrast to the invasive *T. gondii* stage, *Giardia* parasites attach extracellularly to the luminal part of the intestinal cell layer. This physical interaction is thought to modulate the intestinal barrier function as a proposed pathophysiological mechanism of disease, however, the detailed mechanisms remain largely unknown (Kraft et al., 2017; Allain and Buret, 2020; Kraft et al., 2020). The symptomatology and ultimate disease outcomes of giardiasis can be broad, and generally include both asymptomatic carriage as well as various gastro-intestinal complaints such as diarrhea and nausea (Allain and Buret, 2020).

The goal of the present study was to provide protocols for the establishment of 3D intestinal organoid cultures and organoid-derived monolayers (ODM) of various host species with zoonotic relevance for *T. gondii* transmission to omnivores. By minimizing the variation of medium composition and step-by-step instructions we provide a robust protocol for establishment of 3D as well as ODM cultures for murine, human, porcine and avian hosts, as these are important habitats for zoonotic parasites such as *T. gondii* and *Giardia* spp. Additionally, we provide an infection model in murine ODMs that allows studying *T. gondii* and *G. duodenalis* co-infection in a relevant primary intestinal tissue.

## Methods

### General Remarks

A detailed list of all medium components, supplements, reagents, kits and cell lines, including respective suppliers, is provided in **Supplementary Table 1**. Antibody suppliers and dilutions used in the study can be found in **Supplementary Table 2**. Primer sequences are given in **Supplementary Table 3**. For gene and protein nomenclature, we followed the international nomenclature guidelines for the respective species. However, in cases where the same molecule of different species was discussed we exemplary followed the nomenclature for vertebrates.

### Establishment and Culture of 3D Organoids

Human, chicken, mouse and porcine crypts were isolated as described previously (Sato et al., 2011a; Mahe et al., 2015). The isolation and establishment of organoids from the human duodenal specimen were described before  (Kraft et al., 2020) and were approved by the ethical committee of the Charite, Berlin (#EA4-015-13). For porcine samples, duodenal crypts were isolated from a 10-week old piglet (*Sus scrofa*, kindly provided by Svenja Steinfelder, Institute of Immunology, Freie Universität Berlin, animal license T0002/17). Chicken duodenal crypts were isolated from intestine of a 14-day-old female chicken (*Gallus gallus*, kindly provided by Luca Bertzbach & Benedikt Kaufer, Institute of Virology, Freie Universität Berlin, animal license T0245/14). The mouse duodenal sample derived from a female C57/Bl6 mouse from an RKI in-house bred colony (animal license T0173/14).

Intestinal sections were opened longitudinally and the intestinal content was removed by washing with ice-cold PBS. The tissue was cut into 5-mm sections and washed in ice-cold PBS until the suspension remained clear. The tissue fragments were incubated on ice in chelating buffer (PBS containing 2% sorbitol, 1% sucrose, 0.05 mM DTT, 10 mM EDTA, 10 μg/ml Fungin, 10 μg/ml tetracycline and 100 μg/ml gentamicin) for 30 min. After settling down of the fragments, the buffer was removed and the fragments were pipetted up and down 10 times in 3 to 5 ml chelating buffer without EDTA. Supernatant was harvested and collected in a sample tube. This step was repeated six more times. The isolated crypts were pelleted by centrifugation at 300*g* for 5 min, 4°C, and resuspended with Advanced DMEM/F12 supplemented with penicillin/streptomycin (P/S, added as standard supplement for Advanced DMEM/F12). After an additional centrifugation step at 300*g* for 5 min, 4°C, crypts were resupended in 1 ml Advanced DMEM/F12 and mixed 1:2 with Matrigel and seeded in 24-well plates as individual 50-µl droplets, comprising between 50 and 100 crypts. Matrigel was incubated at 37°C for ~30 min to allow polymerization before growth medium was added. The medium was exchanged every 2 to 3 days.

Organoid cultures were passaged every 3 to 7 days following the guidelines by Mahe et al. (2015). Chicken organoids were mechanically disrupted by pipetting up and down with a 200-µl pipette tip for 1 min. Human, mouse and porcine organoids were first enzymatically digested with TrypLE Express (5 min at 37°C) and mechanically disrupted by forcing the suspension through a blunt 18G needle. Cells of all species were then washed with Advanced DMEM/F12 and organoids were re-embedded in Matrigel at a 1:2 ratio.

### Media Conditions

Three-dimensional intestinal epithelial cell cultures are first generated by incubating the crypt tissue in an environment that resembles the intestinal crypt condition. These culture conditions support a high proliferative capacity by maintaining stemness of the intestinal crypt base (referred to as *WERN condition*). These conditions promote growth mainly of so-called spheroids that are stem cell-enriched, three-dimensional spherical structures that can be maintained almost indefinitely (Sato et al., 2011a; Miyoshi and Stappenbeck, 2013; VanDussen et al., 2015). To induce differentiation of spheroids into organoids, i.e., structures containing multiple primary epithelial cell types, 3D spheroids were cultured in medium deficient of several stem cell-enriching factors (referred to as *ERN condition*, leaving out Wnt3a) to transform into differentiated organoids.

3D culture was performed using base medium with supplementation of small molecules and inhibitors as summarized in **Table 1**. Base medium (WERN) consisted of 50% L-WRN-conditioned media [CM, L-WRN ATCC CRL-3276; VanDussen et al. (2019)], 20% R-Spondin1 CM [293T cells stably expressing RSpo1-Fc (Kim et al., 2005), a kind gift from Calvin Kuo, Stanford University], 10% Noggin CM (293T cells stably expressing mNoggin-Fc, a kind gift from Hans Clevers, Utrecht University), 50 ng/ml EGF, 1 mM HEPES, 2 mM GlutaMax, 1× P/S, 1× N2, 1× B27, 1 mM N-acetylcysteine 10 mM nicotinamide, 500 nM A83-01 (TGF-β inhibitor) and 1 μM SB202190 (p38 inhibitor) in Advanced DMEM/F12. Pig organoids were cultured in base organoid medium with additional 10 µM rho-associated, coiled-coil-containing protein kinase 1 (ROCK1)-inhibitor Y-27632, and chicken organoid cultures were supplemented with further 10 µM prostaglandin E2 and 3 μM CHIR99021 (GSK-3 inhibitor). For cryopreservation, organoids from four wells were harvested, washed and resuspended in 1 ml freezing medium consisting of 10% DMSO, 10% fatty acid free BSA in Advanced DMEM/F12 and placed in cryovials. The vials were slowly frozen down at −80°C in a CoolCell LX container for 24 h before long-term storage in liquid nitrogen. Passage number never exceeded 30 passages for all species.

**Table 1:** Medium conditions for organoid culture.

Host	L-WRN CM*(50%)	Rspo1 CM**(20%)	Noggin CM***(10%)	EGF (50 ng/ml)	NAC (1 mM)	NIC(10 mM)	SB-202190 (1 μM)	A83-01 (500 nM)	Y-27632 (10 μM)	CHIR (3 μM)	PGE2 (10 μM)
Three-dimensional organoids											
Mouse	X	X	X	Mouse	X	X	X	X			
Human	X	X	X	Human	X	X	X	X			
Pig	X	X	X	Mouse	X	X	X	X	X		
Chicken	X	X	X	Human + Mouse	X	X	X	X	X	X	X
Organoid-derived monolayers											
All	(Initially)	X	X	X	X	X			(Initially)		

*L-WRN conditioned medium corresponds to 100 ng/ml recombinant Wnt3A.

**R-spondin1 conditioned medium corresponds to 1 µg/ml recombinant R-spondin.

***Noggin conditioned medium corresponds to 100 ng/ml recombinant Noggin.

NAC, N-acetylcysteine; NIC, nicotinamide.

3D differentiation medium (ERN) consisted of 5% R-Spondin-1 CM, 5% Noggin CM, 1 mM HEPES, 2 mM GlutaMax, 1× P/S, 1× N2, 1× B27, 1 mM N-acetylcysteine and 50 ng/ml EGF in Advanced DMEM/F12 for mouse, human and porcine organoids. As the induced differentiation resulted in fast apoptosis in chicken organoids, spheroids were incubated with a differentiation medium consisting of 20% FCS, 2 mM GlutaMax, 1× P/S and 10 µM ROCK1 inhibitor Y-27632 in Advanced DMEM/F12. Mouse, porcine, and chicken organoids were kept in differentiation medium for three days; human organoids for five. Spheroids were cultured in parallel during differentiation experiments to serve as a control, since degradation of the extracellular matrix could also drive differentiation.

ODM medium consisted of 20% R-Spondin1 CM, 10% Noggin CM, 50 ng/ml EGF, 1 mM HEPES, 2 mM GlutaMax, 1× P/S, 1× N2, 1× B27, 1 mM N-acetylcysteine, and 10 mM nicotinamide in Advanced DMEM/F12 for all species.

### Establishment of Organoid-Derived Monolayers (ODMs)

ODMs were established on 0.33 cm^2^ (PET) or 0.6 cm^2^ (polycarbonate) pre-coated transwell filters (0.4 μm pores). For coating, transwell inserts were chilled at −20°C for 20 min. In the meantime, Matrigel was mixed 1:10 with Advanced DMEM/F12. 150 μl were then pipetted into each of the upper compartments of a transwell insert and incubated for at least 16 h at 4°C. Before seeding, the supernatant was removed and the plate was incubated at 37°C for 30 min. After mechanical disruption (as described for passaging) and washing, singularized cells were collected in a single tube in pre-warmed ODM medium with 50% WRN CM supplemented with 10 µM Y-27632 and directly added onto the transwell inserts (approximately 2–3 × 10^6^ cells per cm^2^ transwell surface). The WRN content was reduced to 5% on day 1 and 0% on day 2, following general guidelines described by Moon et al. (2014) and VanDussen et al. (2015). Y-27632 was withdrawn from cultures on day 2 for all species. The medium was subsequently exchanged three times a week.

### Immunofluorescence Assays (IFAs) and Microscopic Analyses

The ODM medium was removed and cells were subsequently fixed with 4% pre-warmed paraformaldehyde (PFA) in PBS (20 min at RT) or −20°C cold methanol (20 min at −20°C), depending on the primary antibody used (**Supplementary Table 2**) and processed within 7 days after fixation. Cells were permeabilized with 0.1 M glycine, 0.2% Triton X-100 in TBS and incubated in blocking buffer consisting of 3% BSA, 1% normal goat serum, 0.2% Triton-X-100 in TBS (50 mM Tris-Cl, pH 7.5, 150 mM NaCl) for 3 h at RT. Primary antibodies were added for incubation at 4°C overnight in blocking buffer and ODMs were washed the next day four times with 0.2% Triton-X100 in TBS. Secondary antibodies and 0.2 µg/ml DAPI or DRAQ5 as nuclear stains were added, kept for 1 h at RT in the dark, followed by three additional washing steps with TBS. The transwell inserts were washed once with deionized water and the filter surfaces with attached monolayers were then cut out from their frames using a scalpel. They were then mounted with Fluoromount-G on glass slides.

The 3D organoids were harvested and washed once in PBS, then incubated in cell-recovery solution for 30 min, followed by an additional wash with PBS, and fixed for 30 min in 4% PFA. Organoids were permeabilized and blocked as mentioned above, incubated for 2 h with Alexa 488-conjugated phalloidin and DAPI in blocking buffer. This was followed by three washing steps in 0.2% Triton-X100 in TBS and organoids were finally mounted with non-hardening IBIDI mounting medium onto glass slides.

Brightfield images were acquired using an Axio Z1 Observer microscope system (Zeiss). Images were contrast-adjusted with Zeiss ZEN software (blue edition). Fluorescence-microscopic images were taken by a Zeiss LSM 780 confocal laser scanning microscope, equipped with Plan-Apochromat 20×/0.8 M27 and C-Apochromat 40×/1.20 water M27 objectives and analyzed with ZEN software (blue edition) and ImageJ version 1.52a (Schneider et al., 2012). Panel composition and annotations were performed using Adobe Illustrator software. Secondary antibody controls are summarized in **Supplementary Figure 5**. All microscopy experiments were performed at least twice unless otherwise stated.

To quantify *T. gondii* load in infected murine ODMs, projections of z-stacks were generated and channels were separated. Number of nuclei of the parasites and host cells were quantified after size separation using the tool “Analyze particles” implemented in ImageJ. Parasite ratio to host cell was calculated by dividing the number of parasites counted by the number of nuclei counted per field. For each timepoint, three replicates were generated and three microscopic fields per filter were scanned per experiment.

### RT-qPCR Analyses

To remove remaining traces of Matrigel, 3D organoids were excessively washed with ice-cold PBS before RNA extraction. RNA was extracted from 3D organoids and ODMs using Direct-zol RNA Microprep kit including an on-column DNase I treatment. RNA was quantified by 260/280 nm absorption measurement, with an Infinite M200 Pro reader (TECAN). The High Capacity RNA-to-cDNA Kit was used for cDNA synthesis with 200 to 500 ng RNA in 20 µl volume per reaction. qPCR was performed with a BioRad C1000 cycler with CFX96 system with a minimum of 5 ng cDNA per reaction. The qPCR included an initial 10 min enzyme activation step at 95°C, followed by 40 cycles of 20 s at 95°C, 30 s at 60°C, and 20 s at 72°C. To verify amplicon specificity, melting curve analysis was performed in CFX Maestro software. The ΔΔCt method was used to calculate relative expression of transcripts to housekeeping transcripts and, in comparison to spheroid or uninfected conditions, respectively.

### Transepithelial-Electric Resistance (TEER) Measurements

To evaluate the barrier integrity and monolayer formation, TEER measurements were conducted. For this, a Millicell ERS-2 Voltohmmeter and a chopstick Ag/AgCl electrode (STX01) were used. For normalization, blank electric resistance (cell-free, coated transwell insert) was subtracted from raw resistance values and standardized for 1 cm² surface area. All measurements were conducted on a preheated 37°C heating block. For TEER measurements 12 to 36 filter inserts per species were analyzed.

### Maintenance of *T. gondii* and *G. duodenalis* Parasites

*T. gondii* type 1 RH strain tachyzoites stably expressing mitochondrial GFP (Thomsen-Zieger et al., 2003), were maintained by continuous passage in confluent monolayers of human foreskin fibroblasts (HFF; BJ-5ta, ATCC CRL-4001) in DMEM supplemented with 2% FBS at 37°C in 5% CO_2_.

*G. duodenalis* WB6 (ATCC 50803) trophozoites were cultured at 37°C in flat-sided 10 ml cell culture tubes in Keister’s modified TYI-S-33 medium (Keister, 1983), supplemented with 10% adult bovine serum (ABS), 100 µg/mL streptomycin & 100 U/ml penicillin, and 0.05% bovine/ovine bile. For passaging, culture tubes were incubated on ice for 30 min to facilitate trophozoite detachment from the surface. Trophozoites were passaged 3 times a week at a ~1:100 ratio. For infection experiments, parasites were passaged the day before infection to guarantee logarithmic growth phase.

### Infection of ODMs

For *G. duodenalis* infection, it was necessary to incubate ODMs with apical TYI-S-33 medium, as trophozoites survival is not supported in DMEM-based medium (Nash, 2019). As TYI-S-33 is a reducing, cysteine-rich medium, the confluent ODMs were incubated with TYI-S-33 medium in the apical compartment the evening before infection to allow equilibration. Before infection, TEER was measured again to ensure proper epithelial integrity. All infection experiments were conducted with TYI-S-33 medium in the apical compartment and ODM medium in the basal compartment.

For infection with *T. gondii*, HFF cells were scraped from T25 cell culture flasks and passed through a 23G blunt needle. To remove cell debris, the supernatant was collected and centrifuged at 100 *g* for 5 min. The pellet was discarded and the supernatant was then transferred to a new centrifuge tube and spun at 300*g* for 10 min. Tachyzoites were counted in a disposable Neubauer counting chamber and their number was adjusted to 1 × 10^8^/ml in DMEM. TYI-S-33 was aspirated from the apical compartment before an appropriate amount of *T. gondii* in 100 μl DMEM was added on top of the monolayer. After 1 h, DMEM was replaced again by TYI-S-33 and ODMs were either left untouched or co-infected with *G. duodenalis* trophozoites.

*G. duodenalis* trophozoites were detached by chilling on ice, counted and subsequently centrifuged at 1,000*g* for 5 min at 4°C. The pellet was resuspended, their number was adjusted to 1 × 10^8^/ml in TYI-S-33 and parasites were added into the ODM-containing transwell inserts.

For the determination of an infectious dose (ID), we assumed a density of 3 × 10^5^ cells per cm^2^ of a transwell filter, a cell number that was determined in previous experiments. For *T. gondii* infection, we added 25 tachyzoites per host cell (ID 25) and for *G. duodenalis* infection we added 3 trophozoites per host cell (ID 3).

### Immunoblotting

Confluent monolayers of HFFs were detached with a cell scraper and pelleted at 500 *g*. Human organoids were harvested and prepared as for IFAs. *T. gondii* tachyzoites and *G. duodenalis* trophozoites were prepared as described above and then pelleted by centrifugation. All cells were then lysed with RIPA-buffer (50 mM Tris-HCl pH 8.0, 1% w/v NP-40, 0.5% w/v sodium deoxycholate, 0.1% w/v SDS, 150 mM NaCl, and 5 mM EDTA) containing cOmplete protease inhibitor cocktail. Protein concentrations were quantified using a Pierce BCA Protein Assay. For immunoblotting, 2× Laemmli sample buffer (4% w/v SDS, 20% v/v glycerol, 0.004% w/v bromophenol blue, and 0.125 M Tris–HCl (pH 6.8) with 10% v/v 2-mercaptoethanol) was added to the samples and boiled for 5 min. SDS-PAGE in Tris-glycine/SDS running buffer and immunoblotting on nitrocellulose membranes followed standard techniques, with 20 µg protein loaded per lane. Total protein on blots was visualized using DB71 staining (Hong et al., 2000) prior to the antibody incubation. HRP signal was detected using ECL Plus Western Blotting Detection Reagents on a Vilber Fusion FX Western blot and chemiluminescence imaging system.

### Statistical Testing

Basic calculations were performed with MS EXCEL 2010 (Microsoft). Figures were plotted in Prism 8.4 (GraphPad). Quantitative results are presented as mean (± 95% CI). Significance was tested by two-way ANOVA with Dunnett’s correction for multiple testing, and p values ≤ 0.05 were considered as statistically significant. Asterisks indicate statistical significance values as follows: * p < 0.05 ** p < 0.01, *** p < 0.001, **** p < 0.0001.

## Results

### Characterization of Three-Dimensional Duodenal Organoid Cultures From Human, Mouse, Pig, and Chicken

The overall aim of this study was to define experimental parameters for the generation and maintenance of intestine-derived organoids from different host species that are robust but at the same time vary minimally in medium composition and culture conditions.

**Figure 1:**
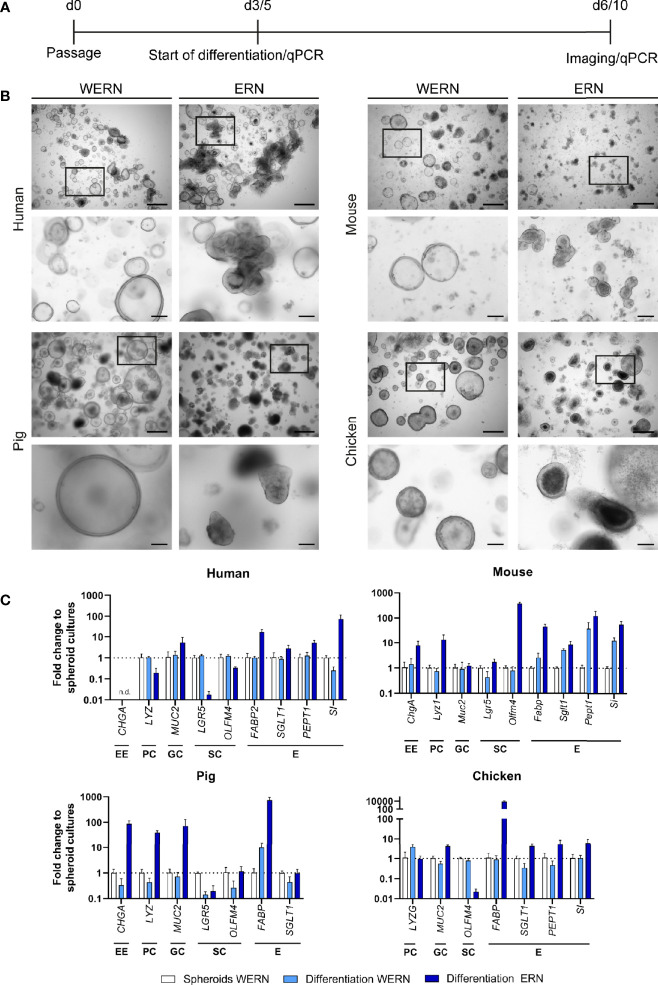
Characterization and differentiation of 3D spheroids/organoids from human, mouse, pig and chicken origin. **(A)** A new spheroid culture was cultured for 3 days (5 days for human) in WERN medium and then spheroids were either maintained in WERN medium or differentiated in ERN medium for further 3 days (human for 5 days). At indicated time points, spheroids/organoids were imaged and/or harvested for RNA extraction and transcriptional analysis by RT-qPCR. **(B)** Representative brightfield microscopic images of spheroids/organoids. Scale bars represent 500 µm (upper panel in each species) and 100 µm (lower panel in each species). **(C)** Quantification of marker genes specific for various intestinal cell types by RT-qPCR. Note that the data are normalized to the transcript abundance of 3/5 day WERN spheroid culture. EE, enteroendocrine cell; PC, Paneth cell; GC, goblet cell; SC, stem cell; E, enterocyte. RT-qPCR experiments show mean (± 95% CI) of ≥ 4 technical replicas of at least two independent biological replicates. n.d., not detectable.

We could establish and maintain human, mouse, pig, and chicken duodenal spheroid cultures by a medium that required only a few general adaptations from previous media conditions defined for human spheroids (**Table 1**, **Figures 1A, B**, WERN). First, we found it essential to provide autologous EGF for optimal growth conditions because of the species-specific variability of the protein sequence in the receptor binding site (**Supplementary Figure 1**). Nevertheless, conveniently, pig EGF could be replaced by mouse EGF and chicken EGF could be replaced by a combination of mouse and human EGF. Of note, growth factors for species other than mouse or human are not readily available, or are prohibitively expensive for high throughput use. Second, to maintain chicken spheroids it was necessary to inhibit glycogen synthase kinase-3 (GSK-3) activity by the inhibitor CHIR99021 in order to maintain stemness conditions by potentiating the β-catenin/Wnt signaling axis as previously described by Li et al. (2018).

Next, we examined the ability of stem cell-enriched spheroid cultures to develop into differentiated epithelial organoids. After incubation in differentiation medium (ERN), spheroids of all species changed in appearance and developed into organoid structures with typical morphological signs of differentiation (**Figure 1B**, ERN). To differing degrees all cultures started to show both budding and the creation of small crypt-like structures that included pronounced apoptotic cell debris accumulating in the inner part of these structures (organoid lumen). Additionally, all cultures developed thicker epithelial layers, and cells grew more columnar in comparison with stem-cell enriched spheroids (WERN) grown in parallel (**Figure 1B**).

To confirm the changes in cell type composition, we analyzed by RT-qPCR the expression of several established marker genes characteristic for the different cell types of the intestinal epithelia, such as enteroendocrine cells, Paneth cells, goblet cells, stem cells and enterocytes (**Figure 1C**, **Supplementary Figure 2**). We compared spheroids (WERN condition) with differentiated organoids (ERN condition), and spheroids that were kept under stem cell-enriching media conditions (WERN) for the same time as organoids in ERN condition, as the degradation of the extracellular matrix may also drive differentiation. Spheroids of all four species differentiated into cell types known to be present in the intestinal epithelium, as judged by the up-regulation of transcripts for respective marker genes. One exception was the lack of enteroendocrine cells in human duodenal organoids (**Figure 1C**). It also appeared that expression of enterocyte markers such as fatty acid-binding protein (*FABP*), peptide transporter 1 (*PEPT1/SLC15A1*) and sucrose isomaltase (*SI*) were particularly elevated in response to the differentiation medium. In contrast, stem cell-associated transcripts generally decreased under differentiation medium conditions, except for mouse organoids.

**Figure 2:**
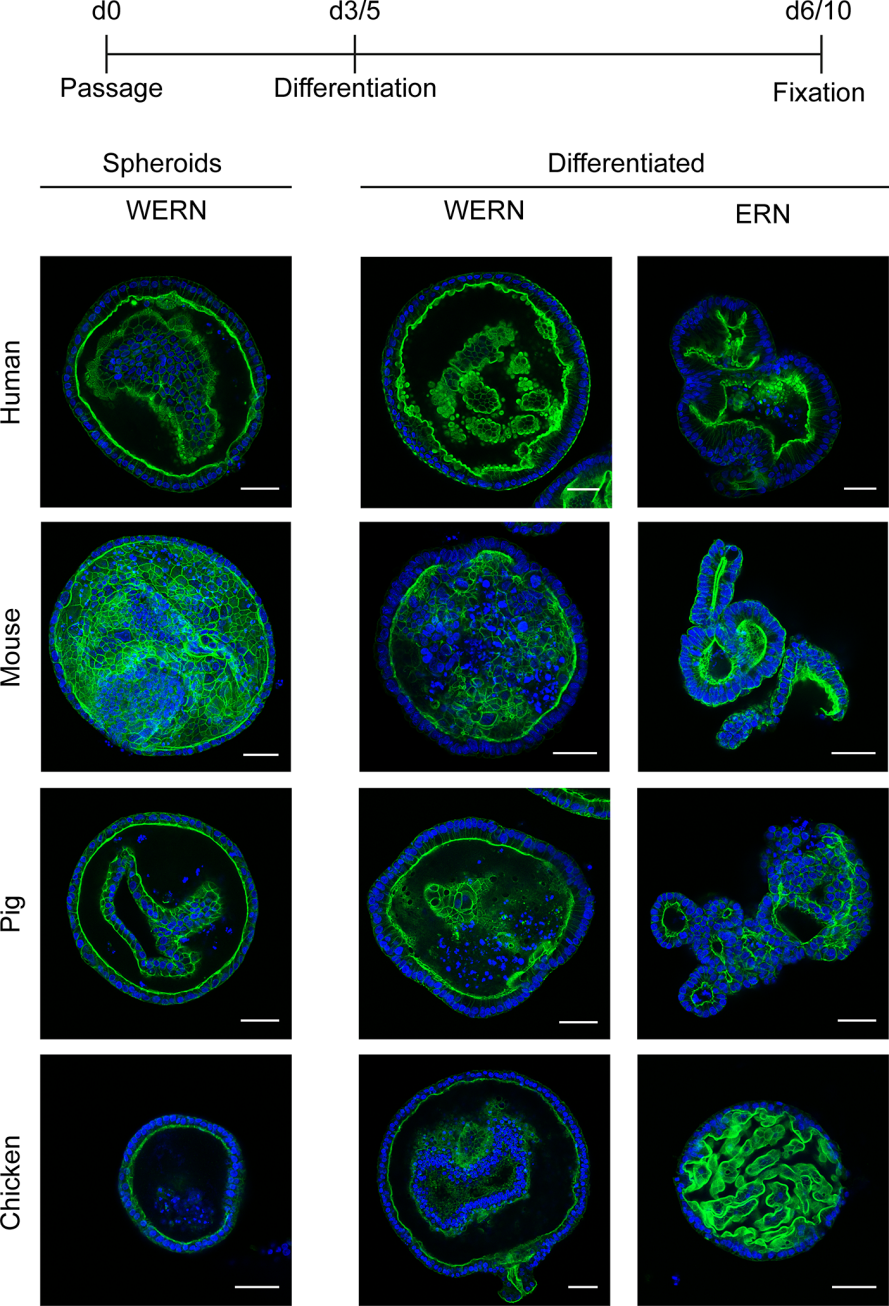
Fluorescence analysis of brush border localization in 3D spheroids/organoids from human, mouse, pig and chicken origin. A new spheroid passage was cultured for 3 days (5 days for human) in WERN medium and then spheroids were either maintained in WERN medium or differentiated in ERN medium for further 3 days (human for 5 days). As a control, 3/5 day spheroids in WERN were also assessed in parallel. Subsequently spheroids/organoids were fixed and F-actin in the microvilli of the luminal brush border was visualized by staining with Phalloidin iFluor-488 (green). Cell nuclei were stained with DAPI (blue). Note, video composition of z-stacks are also provided as **Supplementary Material**. Scale bars represent 50 µm. All experiments were performed twice with similar results.

To characterize the orientation and polarity of the epithelium, we performed fluorescence analysis of spheroids/organoids by labeled phalloidin staining of F-actin to highlight the intestinal brush border (**Figure 2**). As expected, all stages possessed a brush border orientated towards the lumen of the spheroid/organoid structure, thereby exposing the basal side to the surrounding medium. Additionally, under differentiation conditions columnar enlargement of the cells in the organoid walls could be observed.

### Generation of Organoid-Derived, Polarized, Epithelial Monolayers Suitable for Parasite Infection

Given the constraints of limited access to the apical side in 3D organoids for infection, we established conditions for a cellular system that provides easily accessible but functionally separated apical and basolateral compartments. To this end, we seeded singularized 3D spheroid-derived cells onto transwell filters. To start differentiation, we withdrew the stem cell-promoting factors Wnt3a, A83-01 (TGF-beta inhibitor), and SB202190 (p38 inhibitor) from the medium.

**Figure 3:**
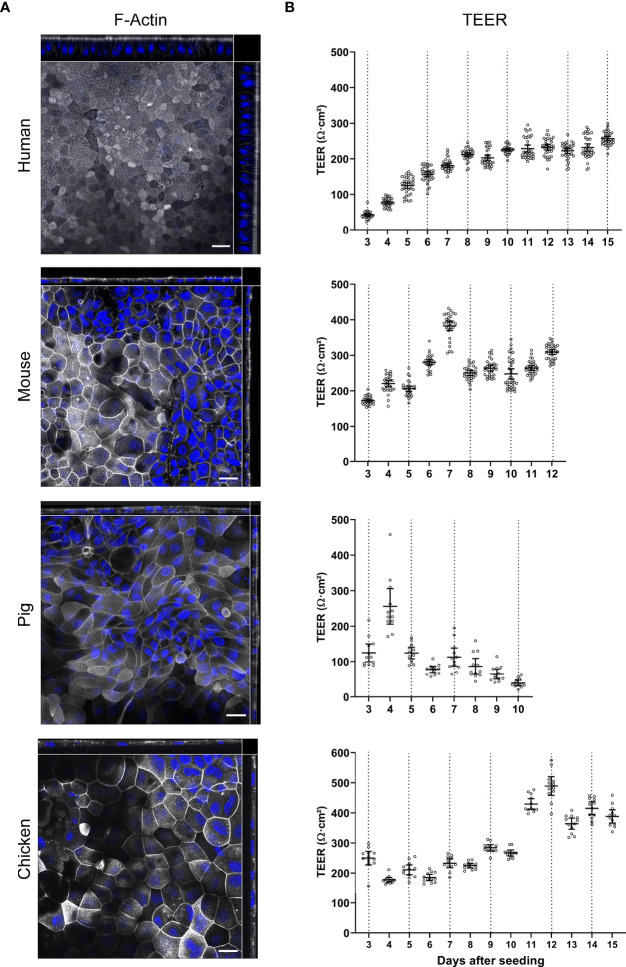
Characterization of TEER development and brush border orientation of ODMs from human, mouse, pig and chicken origin. Spheroids of all four species were seeded on transwell inserts and cultured in differentiation media. **(A)** Representative orthogonal images of z-stacks showing F-actin accumulation in the apical brush border of ODMs. ODMs were fixed after 10 (human), 12 (mouse), 6 (pig) and 15 (chicken) days and F-actin was visualized using phalloidin (white) and nuclei using DAPI (blue). Scale bars represent 20 µm. All experiments were performed twice with similar results. **(B)** ODMs of all four species developed electrophysiologically tight barriers as indicated by TEER analysis. Dashed lines represent medium exchanges. Experiments show mean (± 95% CI) of 12–36 filter inserts per species.

For all four species we could observe that both confluent and polarized epithelial monolayers developed (**Figure 3A**). We recently reported that human ODMs developed a columnar shape with an average thickness of ~20 µm over time (Kraft et al., 2020) (**Figure 3A**, **Supplementary Figure 3**), reflecting polarized epithelial differentiation. In contrast, murine, porcine and chicken ODMs remained comparatively flat, with a thickness of < 10 µm (**Figure 3A**, **Supplementary Figure 3**). Importantly, however, cells of all species were able to generate an electrophysiologically tight epithelium, as indicated by TEER measurements (**Figure 3B**). While the human ODMs reached a plateau at ~220 Ω*cm^2^ after approximately 8 to 10 days, murine, porcine and chicken ODMs formed a tight epithelium already after 3 to 5 days. It is also noteworthy that porcine ODMs collapsed after 6 to 9 days after establishment whereas ODMs of all other species could be maintained for at least 3 weeks (data not shown).

**Figure 4:**
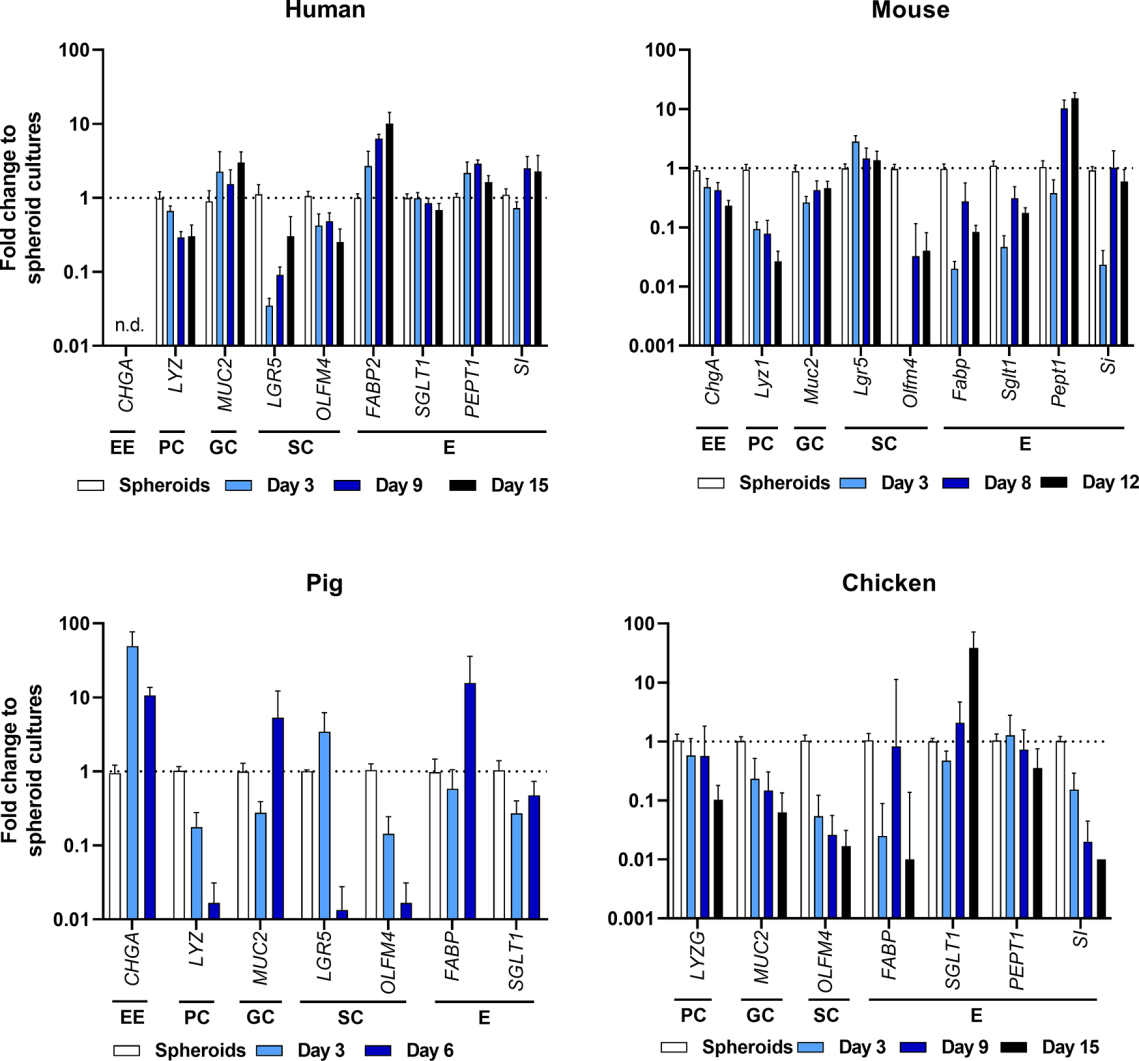
Expression profiling of intestinal cell types by RT-qPCR of ODMs from human, mouse, pig and chicken origin. Indicated markers represent EE, enteroendocrine cells; PC, Paneth cells; GC, goblet cells; SC, stem cells; and E, enterocytes. RT-qPCR experiments show mean (± 95% CI) of ≥ 4 technical replicas of at least two independent biological replicates. n.d., not detectable.

**Figure 5:**
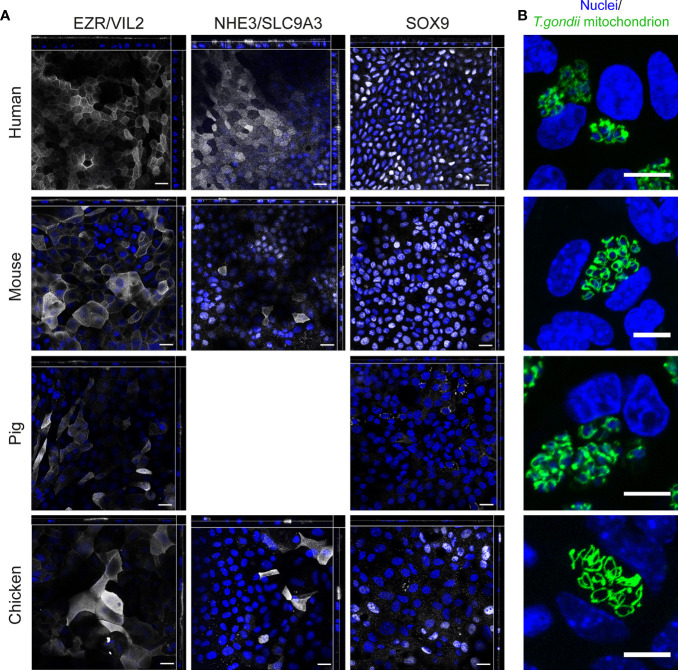
Immunofluorescence analysis of ODMs from human, mouse, pig and chicken origin. **(A)** Orthogonal stacks for brush border protein EZR, enterocyte marker NHE3, and SOX9. ODMs were cultured as described and fixed after 10 (human), 12 (mouse), 6 (pig) and 15 (chicken) days. Note, NHE3 analysis for pig was excluded as suitability of the antibody for IFA staining in this species is questionable (see **Supplementary Figure 6**). Suitability of the SOX9 antibody for IFA staining for pig is also questionable, however, the analysis was included as the antibody target sequences are highly similar between species (see **Supplementary Figure 6**). Representative fluorescent orthogonal stacks of ODMs are shown. Scale bars represent 20 µm. **(B)** Representative confocal image projections of ODMs infected for 48 h with *T. gondii* (distinguished by its GFP-tagged green tubular mitochondria) in TYI-S-33 medium. Scale bar 10µm. All experiments were performed at least twice with similar results.

To compare cell differentiation in ODMs in comparison with the initial spheroid-derived cell cultures, we followed the changes of cell marker transcripts over time. As shown in **Figure 4**, the overall transcriptional pattern changed in all species from crypt-based stem cell/Paneth cell signatures [reflected by lower abundance of Leucine-rich repeat-containing G-protein coupled receptor 5 (*LGR5*), olfactomedin 4 (*OLFM4*), and lysozyme (*LYZ*)] to more villus-like enterocyte/goblet cell signatures [higher abundance of Mucin 2 (*MUC2*) and enterocyte transporters such as *PEPT1*, *FABP*, sodium-glucose linked transporter 1 (*SGLT1*) and *SI*]. However, it is important to note that abundance of specific transcripts differed from species to species, indicating different grades of differentiation (**Figure 4**, **Supplementary Figure 2**). In particular, the murine ODMs revealed still high expression of stem cell markers but also increased Pept1 expression,

To further characterize the cell lineages, we performed IFAs with antibodies directed against the essential brush border formation protein Ezrin (EZR/VIL2), Angiotensin-converting enzyme 2 (ACE2, the SARS-CoV-2 receptor) and sodium–hydrogen exchanger 3 (NHE3/SLC9A3) that are primarily expressed in enterocytes. The results were consistent with the development of both polarized and differentiated ODMs, as EZR and enterocyte markers accumulated at the apical tip of the monolayer cells (**Figure 5A**, **Supplementary Figure 4**). The general picture of a more enterocyte-guided differentiation by our transcriptional analysis was supported by the identification of NHE3- and/or ACE2-positive cells in human, mouse and chicken ODMs. Of note, pig ODMs lacked antibody binding for NHE3 and ACE2, which can also reflect missing conservation of epitopes between species (**Figure 5A**, **Supplementary Figure 4**). We also observed the presence of SRY-Box Transcription Factor 9 (SOX9) in ODMs from human, mouse and chicken, which is an indicator for the presence of proliferative cells in these ODMs (Blache et al., 2004) (**Figure 5A**, **Supplementary Figure 4**).

### Effects of Infection and Co-Infection With *T. gondii* and *G. duodenalis* on Barrier Function of Murine ODMs

The house mouse is an important natural host for *T. gondii* and also the prime experimental host model, not the least due to numerous knock-out mouse strains available that allow detailed studies on host-parasite interaction. In this paper, we therefore focused on the establishment of conditions for infection experiments of murine ODMs. Having a shared interest in the two parasites *T. gondii* and *G. duodenalis*, we also wanted to exploit the challenges given by their different growth conditions in this cellular system to study co-infections. Both parasites infect the same site of the gut, albeit with different modes of infection, and both have a wide and overlapping host range, including mice. However, co-infection studies have been very rarely reported.

We have recently shown that *G. duodenalis* alters the junctional integrity in human ODMs that leads to de-localization and disruption of the junctional complex (Kraft et al., 2020). *T. gondii* has also been proposed to modulate barrier dysfunction in Caco-2 cells (Briceño et al., 2016). We therefore wanted to examine whether similar adverse effects on tight junction integrity could be seen in *T. gondii* infections, alone and also in co-infection with *G. duodenalis*. In the latter case, the presence of tachyzoites might be able to modulate *G. duodenalis*’ effect on barrier function, given the known influence of *T. gondii* on host cell transcription in general (Hakimi et al., 2017).

*G. duodenalis* infection requires incubation of the ODMs with a cysteine-rich medium—Keister’s modified TYI-S-33 medium (Keister, 1983)—that contains bile salts and thereby resembles duodenal conditions. This medium is toxic to commonly-used immortalized cell lines such as Caco-2 cells (Kraft et al., 2017; Nash, 2019), but we have recently shown that these conditions are tolerated by human ODMs (Kraft et al., 2020). We have also shown that incubation of *G. duodenalis* in TYI-S-33 in the apical compartment of transwell inserts supports parasite growth for > 72 h (Kraft et al., 2020) while incubation with DMEM-based media is known to compromise *G. duodenalis* viability (Nash, 2019). As a first step to co-infection, we ensured that both the epithelial monolayers of mouse, pig and chicken, as well as *T. gondii* tachyzoites were tolerant to incubation in TYI-S-33. Incubation with this medium in the apical compartment was tolerated by ODMs of all four species, as determined by analysis of TEER (data not shown). We also observed that the use of TYI-S-33 allowed reproducible replication of RH strain tachyzoites in ODMs of all four species (**Figure 5B**). Quantification of *T. gondii* tachyzoite growth in murine ODMs is shown in **Supplementary Figure 7**.

**Figure 6:**
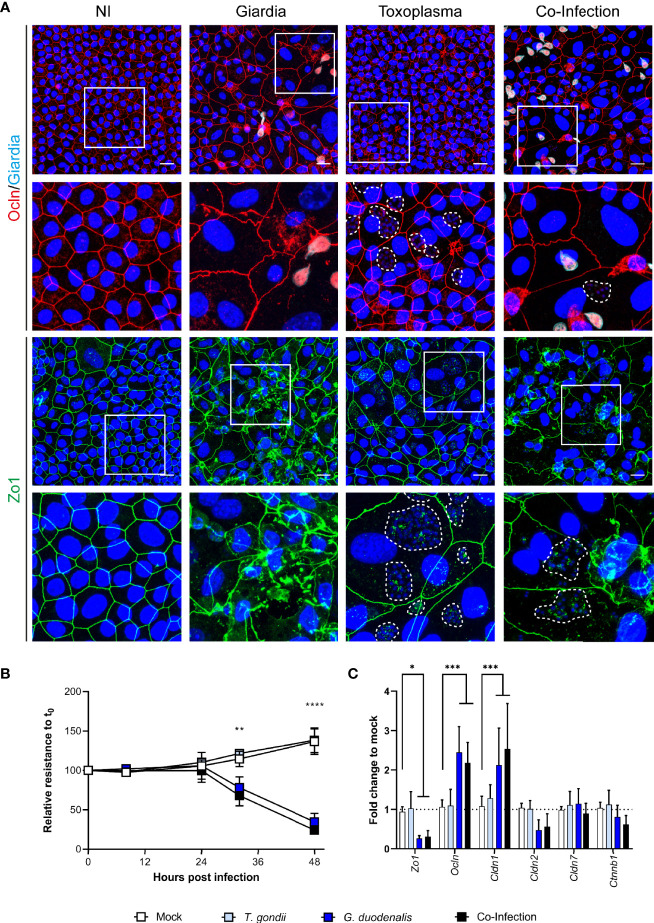
Co-infection of murine ODMs with *T. gondii* and *G. duodenalis*. Murine ODMs were infected with either an ID of 3 (*G. duodenalis* strain WB6 trophozoites) or an ID of 25 (*T. gondii* strain RH tachyzoites), or both and monitored for 48 h post infection. **(A)** Representative projections of immunofluorescence Z-stack images of Zo-1 and occludin of non-infected (NI), *G. duodenalis, T. gondii* and co-infected murine ODMs after 48 h. *T. gondii*’*s* mitochondrial GFP fluorescence was lost due to methanol fixation; instead, dashed lines indicate *T. gondii* vacuoles including parasite nuclei (blue). Scale bars represent 20 µm. All experiments were performed twice with similar results **(B)** TEER monitoring of infected murine ODMs. Data are presented as mean (± 95% CI) of four independent experiments with three filters per experiment. Statistical significance between infection and mock controls was determined using a Two-Way ANOVA with Dunnett’s correction for multiple testing. **p < 0.01, ****p < 0.0001 **(C)** Transcriptional changes of indicated tight junctional components in infected ODMs. Data are presented as mean (± 95% CI) of three independent experiments with three filters per experiment. Statistical significance was determined using a Two-Way ANOVA with Dunnett’s correction for multiple testing. *p < 0.05, ***p < 0.001.

Having established conditions suitable for co-infection, we next designed an experiment using murine ODMs as host system. To this end, ODMs were first infected with *T. gondii* tachyzoites for 1 h and subsequently co-cultured with or without *G. duodenalis* trophozoites for 48 h. As representative protein components of the intra- and intercellular tight junction complex, we choose zona occludens 1 (Zo-1/Tjp1) and occludin (Ocln) and evaluated by IFA whether infection with the parasites alone or in co-infection lead to alterations in barrier integrity (**Figure 6A**). While *T. gondii* showed no apparent effect on the ODMs’ tight junction proteins, *G. duodenalis*’ influence was striking (**Figure 6A**). Infection resulted in severe delocalization and destruction of the tight junctional integrity of murine ODMs. Still, we could identify several cells infected with *T. gondii* in the vicinity of *G. duodenalis* parasites, indicating tachyzoite replication. Overall, co-infection recapitulated the phenotype of the *G. duodenalis* infected condition with no exacerbating or dampening effect by *T. gondii* co-infection (**Figure 6A**). Notably, one report using m-ICc12 cells described a re-localization of occludin at the parasites´ point of entry and co-localization of occludin with the parasite (Weight et al., 2015). In our experiments with murine ODMs, we also observed some staining of tachyzoites in the IFAs with the same antibody (**Figure 6A**). However, Western blot analysis revealed that this reactivity is most likely due to an unrelated *T. gondii* protein cross-reacting with the respective commercial anti-occludin antibody (**Supplementary Figures 8** and **9**).

We also quantified the disturbance of the barrier function using TEER measurements. Using identical IDs as shown in **Figure 6A** *T. gondii* infection alone did not lead to a decrease in epithelial resistance, while the TEER of *G. duodenalis* infected ODMs was significantly deviating from the control conditions after 32 and 48 h post infection (**Figure 6B**). Similar results were obtained for co-infection. RT-qPCR analysis of transcripts of components of tight junctions corroborated these findings. While no significant changes compared to non-infected control conditions could be observed in *T. gondii*-infected ODMs, those containing *G. duodenalis* showed differentially altered tight junction genes *Zo-1* (p < 0.05), occludin (p < 0.001) and claudin-1 (*Cldn1*, p < 0.001, **Figure 6C**). Taken together, in this ODM co-culture system, *G. duodenalis*’ detrimental effect on barrier function is not altered by a concomitant *T. gondii* infection.

## Discussion

Here we provide robust protocols to establish and maintain stem-cell enriched spheroid cultures of four relevant host species that are subsequently used to generate electrophysiologically tight and differentiated ODMs suitable for *T. gondii* infections, alone or together with other relevant intestinal protozoa. We further provide evidence that *T. gondii* infection has, in contrast to *G. duodenalis*, no detrimental effect on TEER and tight junction integrity of murine ODMs. We anticipate that this ODM platform will help to uncover early events in infection of *T. gondii* and other relevant intestinal parasites in the intestinal epithelia in the zoonotic context.

In recent years, the organoid model has been used to overcome limitations of immortalized cell lines, as for example their inability to represent the different cell types of the primary epithelium (Klotz et al., 2012). Beginning with the intestinal adult stem cell-derived mouse organoids (Sato et al., 2009; Sato et al., 2011a), the generation of intestinal organoids has been reported from numerous other species such as mice, humans, cats, dogs, rat, cattle, pigs, birds, and even reptiles (Sato et al., 2011b; Gonzalez et al., 2013; Powell and Behnke, 2017; Chandra et al., 2019; Derricott et al., 2019; Martorelli Di Genova et al., 2019; Ambrosini et al., 2020; Hedrich et al., 2020; Post et al., 2020). Their resemblance to *in vivo* tissue, i.e., the presence of multiple cell types and tissue specific organization, makes organoids more complete and thus superior cellular systems to what most current cell line-based models are able to provide. Consequently, organoids have enabled previously unseen progress in many fields, one of them being infection biology, in particular for bacteria and viruses [reviewed in Hill and Spence (2017) and Artegiani and Clevers (2018)]. The organoid model has enabled advances in the field of parasitology with infection studies on *Cryptosporidium* (Heo et al., 2018; Wilke et al., 2019), *T. gondii* (Derricott et al., 2019; Luu et al., 2019; Martorelli Di Genova et al., 2019), *G. duodenalis* (Kraft et al., 2020) and helminths (Eichenberger et al., 2018; Duque-Correa et al., 2020).

The primary motivation for this study was to develop straightforward protocols for the generation of 3D-organoids and ODMs of different hosts to facilitate the *in vitro* evaluation of host-parasite interactions in the intestinal epithelium (Delgado Betancourt et al., 2019; Hares et al., 2020). As with any other cellular model system, the standardization of reagents, materials and experimental execution is critical in order to provide robust and reproducible results (**Supplementary Tables 2** and **3**). This is even more important for 3D-organoids and ODMs, as small changes in growth and differentiation factors or growth conditions can lead to different cellular compositions. This, in turn, may lead to corresponding differences in parasite behavior. In this context, it must be emphasized that for economic reasons, we used supernatants from cell cultures expressing the three most important (and most expensive) growth factors Wnt3a, R-Spondin1/3 and Noggin, which require careful activity testing and quantification prior to their use to guard from batch-to-batch variation (VanDussen et al., 2019).

Only a few other examples show similar comparisons of organoids from different vertebrate species other than mouse and human to study pathogen interaction in the zoonotic context. Derricott et al. (2019) compared mouse 3D-organoids with those from porcine and bovine origin and compared the morphology as well as selected proteins by mass spectrometry in order to confirm the presence of differentiated cells in the generated cultures (Derricott et al., 2019; Hares et al., 2020). They also showed susceptibility of these 3D organoids to *Salmonella* and *T. gondii* infection, but did neither report quantitative and functional data upon infection nor did they establish monolayer cultures. Culture conditions were different for bovine and porcine cultures, as bovine cultures did not survive in IntestiCult medium (commercial medium optimized to support mouse organoids) alone and required addition of Wnt3a CM. Another group described the formation of porcine ileum 3D cultures and transwell monolayers and used a similar medium containing Wnt3a, as described in the present work for 3D organoids (van der Hee et al., 2018; van der Hee et al., 2020). A further comparative study on 3D cultures from large farm and small companion animals also showed the suitability of WRN CM medium to support growth of organoids from various mammalian species (Powell and Behnke, 2017). Species-specific media components have been extensively described for the mouse and human organoid system (Sato et al., 2011a; VanDussen et al., 2019). For the monolayer system, the requirements are less defined. For example, for mouse and human, the use of 5% WRN CM and 20% FBS has been reported (Moon et al., 2014; VanDussen et al., 2015), whereas Kozuka et al. (2017) report medium requirements without Wnt3a and additional FBS, depending on species and tissue section. Of note, in the murine ODM system, our protocol maintained a high level of stem cell marker expression and induced expression of enterocyte marker Pept1. This might be a result of nicotinamide in the differentiation medium that has been shown to be critical for maintaining prolonged cultures of human organoids (Sato et al., 2011a). How the differentiation status possibly affects infection with *T. gondii* or *G. duodenalis* has not been investigated in the present work. This should be addressed in future studies, including the use of different media conditions as exemplary described above.

For porcine monolayers a medium containing 30% Wnt with additional 20% FBS was used, and measurement of TEER until 72 h after seeding showed an increase of epithelial resistance (van der Hee et al., 2018; van der Hee et al., 2020). However, whether TEER would decline after extended culture times, as observed in our study, was not reported. The observed breakdown of the barrier function in the pig ODMs might be a result of the deprivation of stem cells and the strong epithelial differentiation in these cultures.

Our ODM medium was not supplemented with Wnt3a or FBS, and we have not compared the influence of these factors on the ODMs of the various species. Nonetheless, it also supported ODM culture from chicken that have, to our knowledge, not been reported before. Thus, our protocol provides a reasonable starting point and, if required, may be further optimized.

We have focused on murine, human, porcine and avian models as a starting point for evaluating intestinal interactions with parasites such as *T. gondii* and *Giardia* spp., as these zoonotic parasites are commonly encountered in these host populations. Moreover, while mice are the most studied species as a host for *T. gondii*, pigs, chickens, and cattle are important meat sources for humans. Studying the early phase of infection with *T. gondii* comparatively in these different host organoids might reveal similarities and differences that could lead to new insights on infection routes and early events affecting susceptibility to infection in different species. For instance, the influence of higher temperature (41°C) on the parasitic infection process can be studied with chicken organoids where this temperature is physiological.

Notably, infections initiated with either bradyzoites or oocyst-derived sporozoites, which are the *T. gondii* stages that actually come into contact with the intestinal epithelium in any host (Delgado Betancourt et al., 2019), could be developed using this ODM platform. Most prior investigations of host-pathogen interactions employ tachyzoites, as the other developmental stages are scarce resources. It thus remains an open question whether infections initiated with the different stages results in obvious differences in infection dynamics using intestinal organoids. Furthermore, it is known that the tissue migratory capacity of *T. gondii* varies between strains (Barragan and Sibley, 2002), with type I strains (used in this study) having a better migratory capacity compared to type II and type III strains. The presented models will allow the comparison of infections with *T. gondii* strains of different genetic backgrounds under standardized conditions (infection dose, timing, nutritional composition, etc).

Of course, the absence of microbiota and immune cells in this system has to be taken into account and its pros and cons and the possibilities to supplement the organoid system with these important players have been described recently (Noel et al., 2017; Williamson et al., 2018; Bar-Ephraim et al., 2020; Min et al., 2020). There are other limitations of organoids in general. For example, cell compositions of these cultures will vary not only from host-to-host but also between individuals reflecting e.g. genetic variability, unless this variation is known, comparative studies and data have to be interpreted with caution. Another limitation relates to the lack of standardized culture conditions between laboratories. However, the field is not yet at this stage. Providing a point of departure towards comparability has been a motivation for this work, though. Another limitation relates to the difference in longevity of the differentiated cultures. Although indefinite proliferation is generally achieved under Wnt-supplemented conditions, the differentiated organoids, in particular under monolayer conditions, are less stable in certain species and medium conditions. Not surprisingly, most publications describe monolayer systems used for time frames of 1 to 6 days after seeding (Moon et al., 2014; VanDussen et al., 2015; Kozuka et al., 2017), enough to show appropriate differentiation of the major cell types of the epithelium. Our main goal was to find conditions that provided stable monolayer conditions — that is maintenance of TEER — suitable for “longer” experiments. Our culture conditions indeed provided stable TEER values until 3 weeks after seeding (except for porcine ODMs). Depending on the question, media conditions need to be adapted as described above and by others (Kozuka et al., 2017).

While the dynamics of invasion and dissemination of *T. gondii* in the lamina propria are better studied, the cellular mechanisms of initial epithelial invasion still remain to be elucidated (Delgado Betancourt et al., 2019). Typically, previous infection models merely simulate the architecture of the intestinal epithelium, using intestinal cell lines such as murine m-ICc12 (Rachinel et al., 2004), human Caco-2 (Briceño et al., 2016; Ross et al., 2019), and rat IEC-6 cells (Weight et al., 2015). However, with respect to differentiation-related marker protein expression, these immortalized cell lines often retain a memory of their tissue origin and do not have the potential to reflect the complexity of the epithelium, i.e. the heterogeneity of cell populations found throughout the intestinal crypt-villus axis (Pageot et al., 2000; Balimane and Chong, 2005; Hill and Spence, 2017).

It has been suggested that *T. gondii* moves through the epithelial junctions for transepithelial migration and penetrates the lamina propria without altering paracellular barrier functions such as TEER or the permeability of higher molecular weight fluid-phase markers such as dextran (Barragan and Sibley, 2002; Barragan et al., 2005; Lambert and Barragan, 2010; Weight et al., 2015). Measurements of these parameters during infections were performed over short durations of time [90 min, Barragan et al. (2005); Ross et al. (2019) or 2 h (Weight et al., 2015)] to determine the effect during transmigration. Such short observational time points, however, may be insufficient to show the modulation of the tight junction barrier upon infection and intracellular growth of the parasite. In a study by Briceño et al. (2016), a decrease in TEER after 20 and 24 h of infection in Caco-2 cells was reported, pointing toward a modulation of barrier function during the intracellular growth phase. Moreover, studies on the junctional complex in retinal epithelial cells (Song et al., 2017) or human endothelial HUVEC cells (Franklin-Murray et al., 2020) support a similar TEER effect after a 24 h or 18h time point, respectively. In contrast, several groups have analyzed the distribution of junctional proteins during paracellular transmigration of tachyzoites in short term experiments (up to 6 h) and during intracellular growth with different cellular systems with partly conflicting results. For example, Briceño et al. (2016) used Caco-2 cells and reported the redistribution and decrease of Zo-1 after 24 h post infection (p.i.) whereas studies in MDCK (Barragan et al., 2005), m-ICc12 cells (Weight et al., 2015) and Caco2 or murine brain endothelial cells (Ross et al., 2019) showed no alterations in the distribution of this protein after 2 to 6 h p.i. With the longer time points of these studies in mind, we extended TEER measurements, RT-qPCR, and the IFA of tight junction components to 48 h. Notably, we observed no significant TEER changes or tight junction alterations in ODMs in *T. gondii* single infections. A moderate TEER decrease was observed only at much longer time points (>96h) and required higher parasite doses (ID > 50, data not shown). We have not further investigated whether this was due to cell lysis of the monolayers or other mechanisms. We speculate that the complexity of the ODM system and its proliferative capacity is partly responsible for the observed differences to traditional models based on immortalized cell lines. Understanding these differences will be insightful in future comparative studies using ODMs of different hosts.

As for *T. gondii*, most research focusing on the pathogenicity of *G. duodenalis* is performed using immortalized cell lines. To our knowledge this is the first time *G. duodenalis* infection, and co-infection with *T. gondii*, is reported on murine organoid-derived layers. Changes of the epithelial junctional complexes have also been reported in response to *G. duodenalis* infection (Koh et al., 2013; Ma’ayeh et al., 2018). However, in a similar manner to *T. gondii* infections, most studies are inconsistent and observed this change at very early time points of infection (Chin et al., 2002; Ma’ayeh et al., 2018). The exact mechanisms of tight junction breakdown and subsequent TEER decrease *in vitro* are not fully understood. The activation of caspases is reported to lead to apoptosis (Chin et al., 2002) or to the restructuring of the cytoskeleton *via* MLCK (Bhargava et al., 2015) as the main driver in cell line-derived epithelium. While this is superficially supported with our findings (**Figure 6**), we have described elsewhere that tight junction breakdown occurs *via* a different series of events in human duodenal ODMs (Kraft et al., 2020). ODMs thus have reproduced much closer the situation found *in vivo* as reported by Troeger et al. (2007). Here, we also show differential expression of tight junction components in murine ODMs after *G. duodenalis* infection, which partly differed to the response seen in the human ODM system. This indicates species-specific responses, however, the net effect on tight junction barrier impairment as determined by TEER reduction revealed similar kinetics as compared to the human ODM system. Future studies will be necessary to dissect common and species-specific responses.

In conclusion, we provide straightforward protocols to establish and maintain stem-cell enriched primary intestinal epithelial cultures of four relevant host species and their adaptation to generate differentiated monolayer cultures suitable for *T. gondii* infections or co-infections with other relevant intestinal protozoa. The possibility of expansion of the organoid micro-architecture by additional factors such as microbiota and immune cells may aid in understanding infection and disease. In addition, the organoid system is amenable for genetic engineering which might help to identify cell type targeted pathogenic effects. In future studies these systems are therefore suitable to discover host-specific or common factors important for host-parasite relationship in the relevant primary intestinal epithelial tissue.

## Data Availability Statement

The original contributions presented in the study are included in the article/**Supplementary Material**. Further inquiries can be directed to the corresponding author.

## Ethics Statement

The isolation and establishment of organoids from the human duodenal specimen were described before (Kraft et al., 2020) and were approved by the ethical committee of the Charité, Berlin (#EA4-015-13). The donors of human material provided their written informed consent to participate in this study. For porcine samples, duodenal crypts were isolated from a 10-week old piglet (Sus scrofa, kindly provided by Svenja Steinfelder, Institute of Immunology, Freie Universitat Berlin, animal license T0002/17). Chicken duodenal crypts were isolated from intestine of a 14-day-old female chicken (Gallus gallus, kindly provided by Luca Bertzbach & Benedikt Kaufer, Institute of Virology, Freie Universitat Berlin, animal license T0245/14). The mouse duodenal sample derived from a female C57/Bl6 mouse from an RKI in-house bred colony (animal license T0173/14). The animal studies were reviewed and approved by the Landesamt für Gesundheit und Soziales (LAGeSo) Berlin.

## Author Contributions

Study concept and design: all authors. Experiments and analysis of data: DH and ED-B. Interpretation of data: all authors. Drafting the manuscript: all authors. Critical revision of the manuscript for important intellectual content: all authors. All authors contributed to the article and approved the submitted version.

## Funding

Financial support was provided by the Deutsche Forschungsgemeinschaft, DFG *via* GRK 2046 Parasite Infections to TA, FS, and CK. DH and ED-B were funded by GRK2046. Work by TA, FS, and CK cited is supported by the Robert Koch-Institute. FS is a also a member of IRTG 2029 (supported by the German Research Council, DFG), and of TOXOSOURCES (supported by funding from the European Union’s Horizon 2020 Research and Innovation programme under grant agreement No 773830: One Health European Joint Programme).

## Conflict of Interest

The authors declare that the research was conducted in the absence of any commercial or financial relationships that could be construed as a potential conflict of interest.

## Supplementary Material

Supplementary Video 1 Differentiated mouse organoid.

Supplementary Video 2 Differentiated pig organoid

Supplementary Video 3 Differentiated chicken organoid.

Supplementary Video 4 Differentiated human organoid.
