# Supplementary material for: Harmonization of Protocols for Multi-Species Organoid Platforms to Study the Intestinal Biology of Toxoplasma gondii and Other Protozoan Infections
Source: Front Cell Infect Microbiol. 2021 Feb 22;10:610368. doi: 10.3389/fcimb.2020.610368 (PMC7937733; doi:10.3389/fcimb.2020.610368)
Supplement: Supplementary file 1 [file DataSheet_1.pdf]

Supplementary Materials:

Supplementary Table 1. Resources and Materials

Resources	Source	Identifier
**Growth factors and supplements**		
Recombinant Human EGF	Peprotech	AF-100-15
Recombinant Murine EGF	Peprotech	315-09
N-Acetyl-L-cysteine	Sigma-Aldrich	A9165
Nicotinamide	Sigma-Aldrich	N0636
A 83-01	Sigma-Aldrich	SML0788
SB 202190	Cayman chemicals	10010399-10
B-27 Supplement	Gibco	17504044
N-2 Supplement	Gibco	17502048
HEPES (1M)	Gibco	15630056
GlutaMAX Supplement	Gibco	35050038
Penicillin/Streptomycin (100x)	Capricorn scientific	PS-B
Advanced DMEM/F-12	Gibco	12634028
Y-27632 (hydrochloride)	Cayman chemicals	10005583
Prostaglandin E2	Tocris	2296
CHIR-99021	Biozol	TGM-T2310
Matrigel	Corning	734-0269
**Commercial Kits**		
Direct-zol RNA Microprep	Zymo research	R2062
High Capacity RNA-to-cDNA Kit	Applied Biosystems	4387406
ECL Plus Western Blotting Detection Reagents	GE Healthcare	RPN2232
Pierce BCA Protein-Assay	Thermo Fisher Scientific	23225
**Reagent**		
Fetal bovine serum	Gibco	Lot-42G6185K
HI Bovine Serum (ACS)	Gibco	Lot-1954370
Bovine/ovine Bile	Sigma-Aldrich	B8381
TRI Reagent	Zymo Research	R2050-1-200
TrypLE Express Enzym (1X)	Gibco	12605010
Maxima SYBR Green qPCR Master Mix (2X)	Thermo Fisher Scientific	K0223
Cell recovery solution	Corning	11543560
Bovine Serum Albumin	Carl Roth	8076.5
Bovine Serum Albumin (fatty acid free)	Sigma-Aldrich	A7030-100G
IBIDI mounting media	IBIDI	50001
Fluoromount G	Southern Biotech	0100-01
D-Sorbitol	Carl Roth GmbH	6213.1
D-Sucrose	Carl Roth GmbH	4621.2
1,4 Dithiotreit (DTT)	Carl Roth GmbH	6908.1
EDTA pH 8,0 (0,5 M)	Applichem	A4892
Fungin - Antifungal Reagent	Invivogen	ant-fn-1
Tetracycline, Hydrochloride	Merck Millipore	58346
Gentamicin Solution	Capricorn Scientific	GEN-10B
Paraformaldehyde	Carl Roth GmbH	0335.1
Methanol	Carl Roth GmbH	AE71.2
Glycine	Carl Roth GmbH	3187.3
Triton X-100	Sigma-Aldrich	T8787
Normal goat serum	Thermo Fisher Scientific	16210072
Tween 20	Carl Roth GmbH	9127.1
4’,6-Diamidino-2-phenylindole dihydrochloride (DAPI)	Sigma-Aldrich	D9542
DRAQ5	Abcam	ab108410
TRIS-HCl	Carl Roth GmbH	9090.3
cOmplete, EDTA-free Protease Inhibitor Cocktail	Roche diagnistics GmbH	04693132001
Nonidet P-40	Sigma-Aldrich	N-6507
Deoxycholic Acid	Sigma-Aldrich	D2510
Sodium dodecyl sulphate (SDS)	Carl Roth GmbH	CN30.3
Sodium chloride	Sigma-Aldrich	71379
Glycerol	Carl Roth GmbH	3783.2
Bromophenol blue	GE Healthcare	17-1329-01
2-Mercaptoethanol	Sigma-Aldrich	M-3148
Direct Blue 71	Sigma-Aldrich	212407
**Cell lines**		
Human foreskin fibroblasts, immortalized	ATCC	CRL-4001

L-WRN	Miyoshi and Stappenbeck (2013), ATCC	CRL-3276
293T cells stably expressing RSpo1-Fc	Kim et al. (2005)	N/A
293T cells stably expressing mNoggin-Fc	Bartfeld et al. (2015)	N/A
Hybridoma cell line expressing antibody against ZO-1 /TJP1	Developmental Studies Hybridoma Bank	R26.4C
**Parasites**		
Toxoplasma gondii RH βmGFP	Thomsen-Zieger et al. (2003)	N/A
Giardia duodenalis WB6	ATCC	50803
**Other**		
Cell Culture Insert, 0.6 cm², Polycarbonate, 0.4 µm Pores	Merck Millipore	PIHP01250
Cell culture insert, 0.3 cm², PET, 0.4 µm Pores	Corning	353095
Nunclon™Cell culture tubes	Thermo Fisher Scientific	734-2068
C-Chip Disposable Hemocytometer	Kisker Biotech	M-NZ
CoolCell LX	Corning	CORN432004
Nitrocellulose membrane	Thermo Fisher Scientific	10600003
**Software**		
MS Excel 2010	Microsoft	N/A
GraphPad Prism 8.1.2	GraphPad Software	N/A
ImageJ 1.52a	NIH, Schneider et al. (2012)	N/A
CFX Maestro	Biorad	N/A
Adobe Illustrator	Adobe Inc.	N/A
Geneious Prime	Biomatters Inc.	N/A
Zen Software	Carl Zeiss	N/A

Supplementary Table 2. Antibodies and dilutions

Antibodies			
	**Source**	**Identifier**	**Dilution**
**Phalloidin iFluor 488**	Abcam	ab176753	1:1,000
**Phalloidin Alexa Fluor 546**	Invitrogen	A22283	1:300
**Phalloidin iFluor 647**	Abcam	ab176759	1:1,000
**Anti-E-Cadherin, mouse**	BD Biosciences	610182	1:100
**Anti-Occludin, rabbit***	Thermo Fisher Scientific	71-1500	1:300
**Anti-Occludin, rat**	A kind gift from Jerrold R. Turner (Department of Pathology, The University of Chicago, Chicago, Illinois; and Department of Pathology & Division of Gastroenterology, Hepatology and Endoscopy, Brigham and Women's Hospital, Boston, Massachusetts)	N/A	1:10
**Anti-ZO-1, rat***	In-house produced hybridoma supernatant/ Developmental Studies Hybridoma Bank, Iowa	R26.4C	undiluted
**Anti-Lysozyme, Rabbit**	DAKO	A009902	1:300
**Anti-ACE2, goat**	R&D Systems	AF933	1:300
**Anti-Ezrin, mouse**	Abcam	ab4069	1:500
**Anti-NHE3, rabbit**	Novus Biologicals	NBP1-82574	1:300
**Anti-SOX9, rabbit**	Merck Millipore	AB5535	1:300
**Anti-CHGA, goat**	Santa Cruz Biotechnology	sc-1488	1:100
**Anti-ACTB, rabbit**	Cell Signaling	4967S	1:1,000
**Goat Anti-Rabbit Cy5**	Jackson Immuno Research	111-175-144	1:500
**Goat Anti-Mouse Cy5**	Jackson Immuno Research	115-175-146	1:500
**Donkey Anti-Goat Cy5**	Jackson Immuno Research	705-175-147	1:500
**Goat Anti-Rat Alexa 488**	Abcam	ab150157	1:500
**Goat Anti-Rat HRP**	Cell Signaling	7077S	1:3,000
**Goat Anti-Rabbit HRP**	Cell Signaling	7074S	1:3,000

* Antibodies were used with methanol fixation

Supplementary Table 3. RT-qPCR Primer

	Target	Forward	Reverse	Reference/ Primer Bank ID*
**MURINE**				
**Houskeeping**	**Gapdh**	AGGTCGGTGTGAACGGATTTG	TGTAGACCATGTAGTTGAGGTCA	**6679937a1**
**Stem cell**	**Lgr5**	CCTACTCGAAGACTTACCCAGT	GCATTGGGGTGAATGATAGCA	**6753842a1**
	**Olfm4**	CAGCCACTTTCCAATTTCACTG	GCTGGACATACTCCTTCACCTTA	**71892419c1**
	**Sox9**	AGTACCCGCATCTGCACAAC	ACGAAGGGTCTCTTCTCGCT	**165932320c1**
**Enterocyte**	**Si**	ACATTTGCTGGTTCGGGACA	CGCCCCTACCAAAGGCATAC	
	**Fabp2**	GTGGAAAGTAGACCGGAACGA	CCATCCTGTGTGATTGTCAGTT	**6679737a1**
	**Sglt1 (SLC5A1)**	ATGCGGCTGACATCTCAGTC	ACCAAGGCGTTCCATTCAAAG	**31543739a1**
	**Pept1 (SLC15A1)**	CCGGCACACCCTTCTAGTG	TGGCGTTGTGACTGGTGAC	**11078526a1**
**Enteroendocrine cell**	**ChgA**	ATCCTCTCTATCCTGCGACAC	GGGCTCTGGTTCTCAAACACT	**6680932a1**
**Paneth cell**	**Lyz1**	GAGACCGAAGCACCGACTATG	CGGTTTTGACATTGTGTTCGC	**7305247a1**
**Goblet cell**	**ClCa1**	CTAAACATCCGGTCTGCTAGACT	ACCCGTGCGTACACAATCATC	**32964827a1**
	**Muc2**	GCTGACGAGTGGTTGGTGAATG	GATGAGGTGGCAGACAGGAGAC	
**Progenitor**	**Tff3**	TTGCTGGGTCCTCTGGGATAG	TACACTGCTCCGATGTGACAG	**6755773a1**
	**Atoh1**	GAGTGGGCTGAGGTAAAAGAGT	GGTCGGTGCTATCCAGGAG	**6680742a1**
**Junctions**	**Zo1**	GCCGCTAAGAGCACAGCAA	TCCCCACTCTGAAAATGAGGA	**6678355a1**
	**Cldn1**	GGGGACAACATCGTGACCG	AGGAGTCGAAGACTTTGCACT	**7710002a1**
	**Cldn2**	CAACTGGTGGGCTACATCCTA	CCCTTGGAAAAGCCAACCG	**7710004a1**
	**Cldn7**	GGCCTGATAGCGAGCACTG	GTGACGCACTCCATCCAGA	**8393144a1**
	**Occludin (Ocln)**	TTGAAAGTCCACCTCCTTACAGA	CCGGATAAAAAGAGTACGCTGG	**6679162a1**
	**β-Catenin (Ctnnb1)**	ATGGAGCCGGACAGAAAAGC	CTTGCCACTCAGGGAAGGA	**6671684a1**
**PORCINE**				
**Houskeeping**	**Gapdh**	ATCCTGGGCTACACTGAGGAC	AAGTGGTCGTTGAGGGCAATG	**Gonzalez et al. (2013)**
**Stem cell**	**Lgr5**	CCTTGGCCCTGAACAAAATA	ATTTCTTTCCCAGGGAGTGG	**Gonzalez et al. (2013)**
	**Olfm4**	GTCAGCAAACCGGCTATTGT	TGCCTTGGCCATAGGAAATA	**Gonzalez et al. (2013)**
	**Sox9**	CGGTTCGAGCAAGAATAAGC	GTAATCCGGGTGGTCCTTCT	**Gonzalez et al. (2013)**
**Enterocyte**	**Fabp2**	CCGGCAAATACCAAGTACAGA	GCCCCTTCTCCCCAGTCAGGGTCTCC	**Gonzalez et al. (2013)**
	**Sglt1**	GCAGCTGTCTTCCTACTTGC	GCAAACTCGGTAATCATACGG	**Gonzalez et al. (2013)**
**Enteroendocrine cell**	**ChgA**	GACCTCGCTCTCCAAGGAGCCA	TGTGCGCCTGGGCGTTTCTT	**Gonzalez et al. (2013)**
**Paneth cell**	**Lyz**	GGTCTATGATCGGTGCGAGT	AACTGCTTTGGGTGTCTTGC	**Gonzalez et al. (2013)**
	**Dll4**	TCATCATCGAAGCTTGGCAC	GCGCTTCTTGCATAGACGTG	**Gonzalez et al. (2013)**
**Goblet cell**	**Muc2**	GGCTGCTCATTGAGAGGAGT	ATGTTCCCGAACTCCAAGG	**Gonzalez et al. (2013)**
**Progenitor**	**Atoh1**	CACGGGCTGAACCACGCCTT	GGTACCCGCGCTTGCTTCGT	**Gonzalez et al. (2013)**
**CHICKEN**				
**Houskeeping**	**GAPDH**	TGTGACTTCAATGGTGACAGC	GCTATATCCAAACTCATTGTCATACC	**Forder et al. (2012)**
	**ACTB**	CAACACAGTGCTGTCTGGTGGTA	ATCGTACTCCTGCTTGCTGATCC	**Chen et al. (2015)**
**Stem cell**	**OLFM4**	GACTGGCTCTCTGGATGACC	AGCGTTGTGGCTATCACTTG	**Li et al. (2018)**
	**SOX9**	GTGGATGTGGCTCCCTACCC	GGTACTGGTCAGCCAGCTTC	**Nakata et al. (2013)**
**Enterocyte**	**SI**	TCAAATTCCCTACGATGTCCAA	AACAAGAGCCGGTAACCCAGTA	**Yadgary et al. (2011)**
	**FABP2**	AAAGATAATGGAAAAGTACTCACAGCAT	CCTTCGTACACGTAGGTCTGTATGA	**Chen et al. (2015)**
	**SGLT1**	TGGCGGGCTTCTACCGCAGCGAG	CCCGGTAGGTCACCAGTCCCCAG	**Gal-Garber et al. (2000)**
	**PEPT1**	CCCCTGAGGAGGATCACTGTTGGCA	CAAAAGAGCAGCAGCAACGA	**Yadgary et al. (2011)**
**Paneth cell**	**LYZG**	CACGCTGGCAAAATACTGAAG	TTCCCAACACCAGCATTGTAG	**Wang et al. (2019)**
**Goblet cell**	**MUC2**	GCCTGCCCAGGAAATCAAG	CGACAAGTTTGCTGGCACAT	**Chen et al. (2015)**
**Progenitor**	**TFF2**	GCTGTAGCCCTCATCAGCTC	TGTGACTTCAATGGTGACAGC	**Forder et al. (2012)**
	**ATOH1**	GCGGATGCACGGGCTGAACC	GCAGCACACGCCCAGCCAGC	**Mulvaney et al. (2015)**
**HUMAN**				
**Houskeeping**	**GAPDH**	CTCCTGTTCGACAGTCAGCC	CCCAATACGACCAAATCCGTTG	**Kraft et al. (2020)**
**Stem cell**	**LGR5**	GAGTTACGTCTTGCGGGAAAC	TGGGTACGTGTCTTAGCTGATTA	**24475886c3**
	**OLFM4**	ACTGTCCGAATTGACATCATGG	TTCTGAGCTTCCACCAAAACTC	**325053703c2**
	**SOX9**	AGCGAACGCACATCAAGAC	CTGTAGGCGATCTGTTGGGG	**182765453c1**
**Enterocyte**	**SI**	CTGCATTTGAAAGAGGACAGC	ACTCTGCTGTGGAAGTCCTGA	**VanDussen et al. (2015)**
	**FABP2**	ATGGCGTTTGACAGCACTTG	TCAGTTCCGTCTGCTAGATTGTA	**194097324c1**
	**SGLT1**	AGGCTATGACGCCTTCATGG	GGCCCTTGGAGTGTAGCATT	**372622381c3**
	**PEPT1**	GGCTGTAGCCCTGATTGTGT	TGGCAAAACCGATGCACTTG	**Kraft et al. (2020)**
**Enteroendocrine cell**	**CHGA**	AGAATTTACTGAAGGAGCTCCAAG	TCCTCTCTTTTCTCCATAACATCC	**VanDussen et al. (2015)**
**Paneth cell**	**LYZ**	GGTTACAACACACGAGCTACAAAC	AGTTACACTCCACAACCTTGAACA	**VanDussen et al. (2015)**
	**DLL4**	ATTGCCACGGAGGTATAAGGC	TTCACAGTCTGTCCGGTTCC	**Kraft et al. (2020)**
**Goblet cell**	**CLCA1**	TGGTTGCTGAGTCTACTCCTC	TCGTCAAATACTCCCCATCGT	**116006947c2**
	**MUC2**	AGGATCTGAAGAAGTGTGTCACTG	TAATGGAACAGATGTTGAAGTGCT	**VanDussen et al. (2015)**
**Progenitor**	**TFF3**	CCAAGCAAACAATCCAGAGCA	GCTCAGGACTCGCTTCATGG	**16755844a1**
	**ATOH1**	CCTTCCAGCAAACAGGTGAAT	TTGTTGAACGACGGGATAACAT	**4885074c1**

*Primerbank IDs can be traced at https://pga.mgh.harvard.edu/primerbank/index.html as published by Wang et al. (2012).

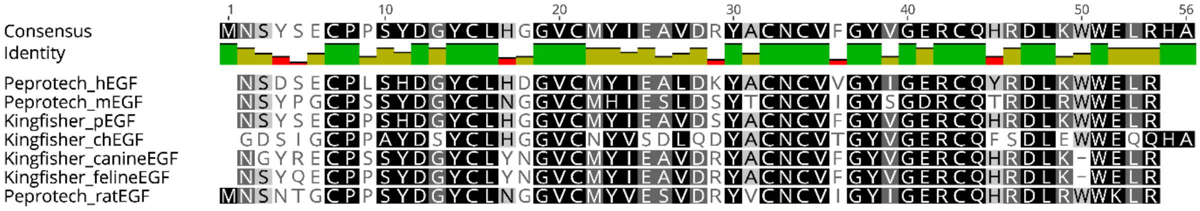

Supplementary Figure 1. Amino acid sequences of commercially available recombinant epithelial growth factor. Sequences show highly variable protein sequence in the receptor binding side. In our hands pig EGF could be replaced by mouse EGF. Additionally, chicken EGF could be replaced by a mixture of human and mouse EGF.

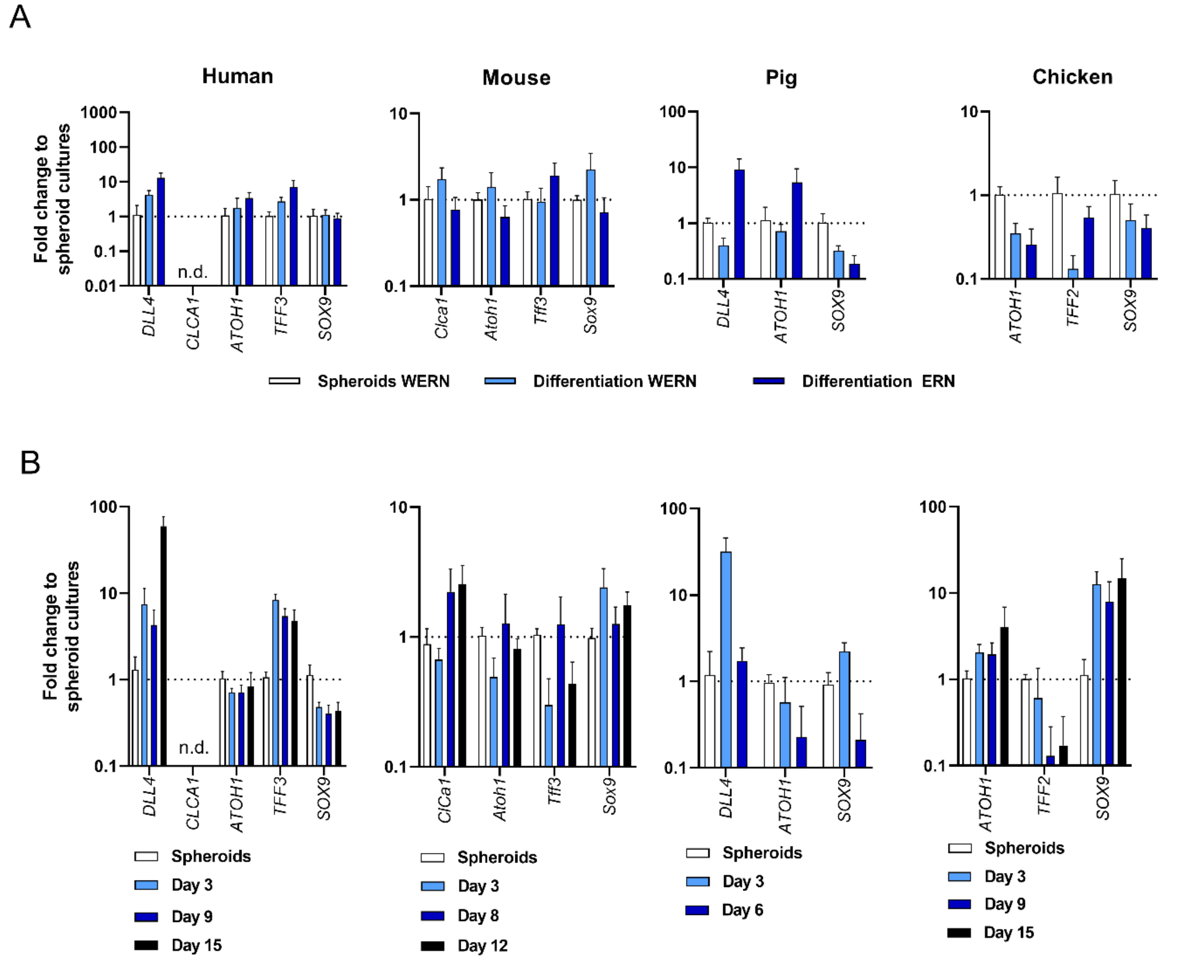

Supplementary Figure 2. Additional intestinal marker genes tested by quantitative RT-PCR. (A) shows additional results of 3D organoid samples while (B) shows results of ODMs. The abundance of specific transcripts differed from species to species, indicating different grades of differentiation. Enhanced transcription of Delta-like ligand 4 (*DLL4*) inhibits hairy and enhancer of split-1 (*HES1*)-induced differentiation into secretory cell progenitors, marked by Atonal BHLH Transcription Factor 1 (*ATOH1*). Chloride channel accessory 1 (*CLCA1*) is primarily expressed by Goblet cells and has a role in loosening of mucus layers. Trefoiled factor 2/3 (*TFF2/3*) are markers of goblet cells that may play a role in mucosal maintenance and repair. SRY-Box Transcription Factor 9 (*SOX9*) in intestines is primarily found in proliferative areas of the crypts. RT-qPCR experiments show the means (± 95% CI) of ≥ 4 technical replicates of two independent biological replicates. n.d. = not detectable.

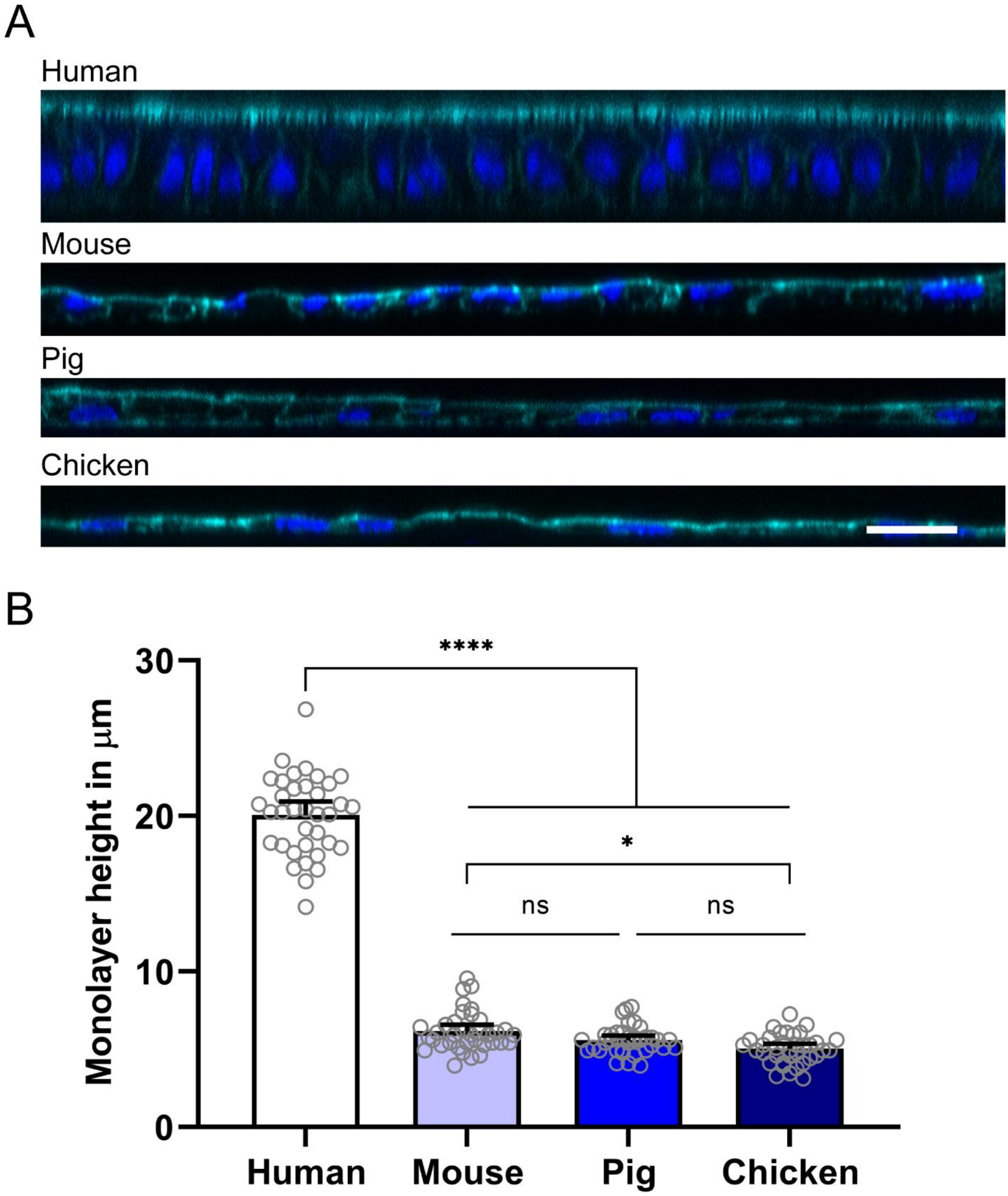

Supplementary Figure 3. Quantification of ODM height. ODM were imaged by confocal microscopy and orthogonal projections were made from z-stacks. (A) Exemplary images of the orthogonal stacks used for the analysis. Scale bar indicates 20 µm. (B) Quantification of the cell height for all species. Height was assessed of three independent ODMs per species. Four different orthogonal stacks per transwell filter were analyzed in ImageJ. Of every orthogonal image, three measurements were performed. Graph shows mean (± 95% CI) of a total of 36 measurements of three independent ODMs per species. Statistical significance was determined using a Two-Way ANOVA with Tukey's correction for multiple testing. * $p < 0.05$, **** $p < 0.0001$

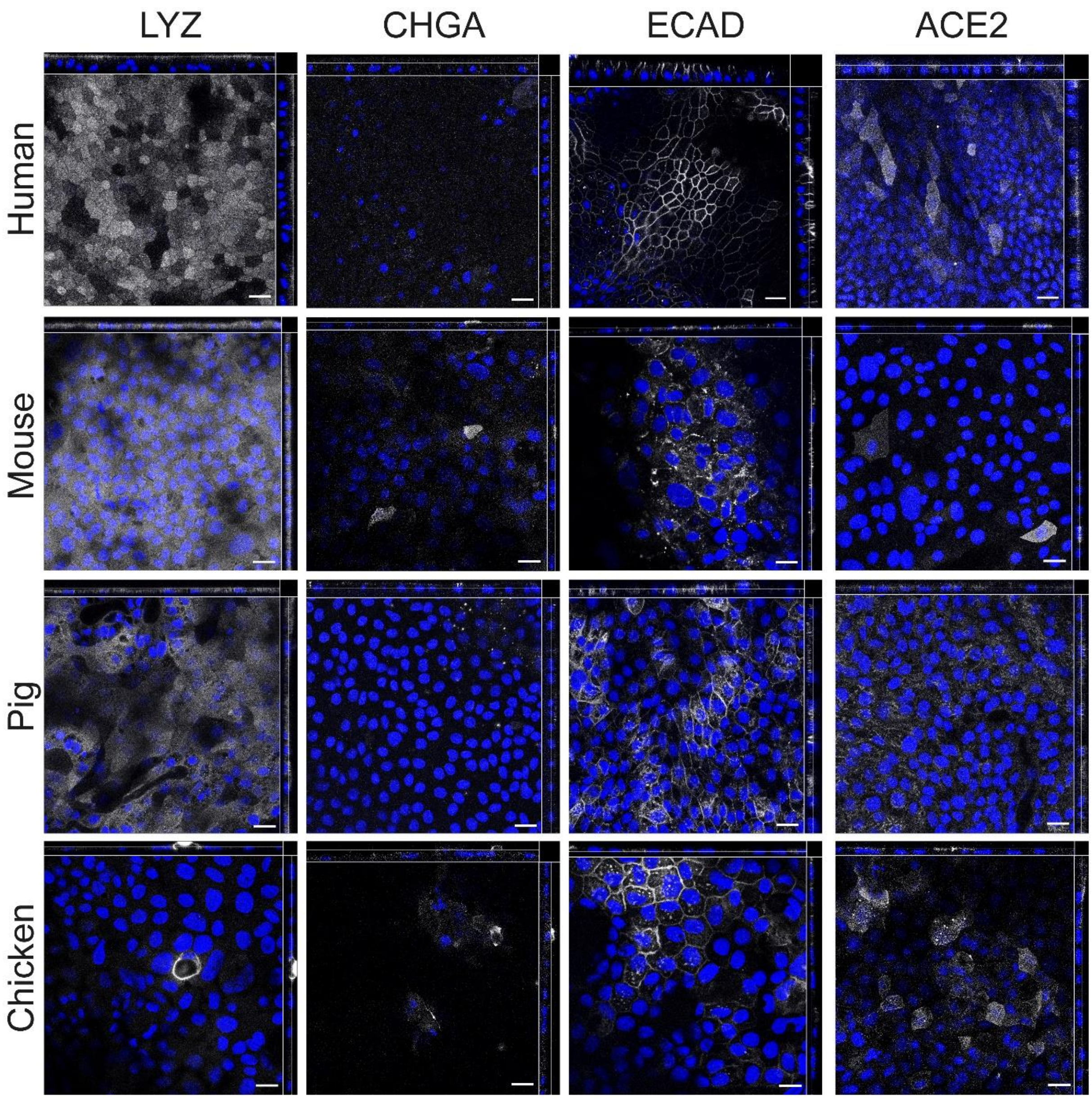

Supplementary Figure 4. Additional immunofluorescence analysis of representative fluorescent orthogonal stacks of organoid-derived monolayers (ODMs). The absence of Chromogranin A (CHGA, a marker of enteroendocrine cells) and presence of Lysozyme [Lyz, usually found in Paneth cell granules, but also produced by small intestinal enterocytes (Wells et al., 2017)] in human ODMs is shown as supported by the RT-qPCR data. CHGA-positive cells are detected in chicken and mouse ODMs. Evenly distributed LYZ positive signal similar to human ODMs was detected in mouse and pig, whereas a distinct signal in Lyz-positive cells was detected in chicken ODMs. E-Cadherine (ECAD/CDH1)-positive staining highlights presence of adherens junctions. ACE2 staining marks enterocytes. Note, lack of antibody binding to ODMs other than mouse and human can also reflect missing conservation of epitopes between species (see amino acid alignments of epitopes recognized by the antibodies in Supplementary Figure 6). All experiments have been repeated twice with similar results. Scale bars represent 20 µm.

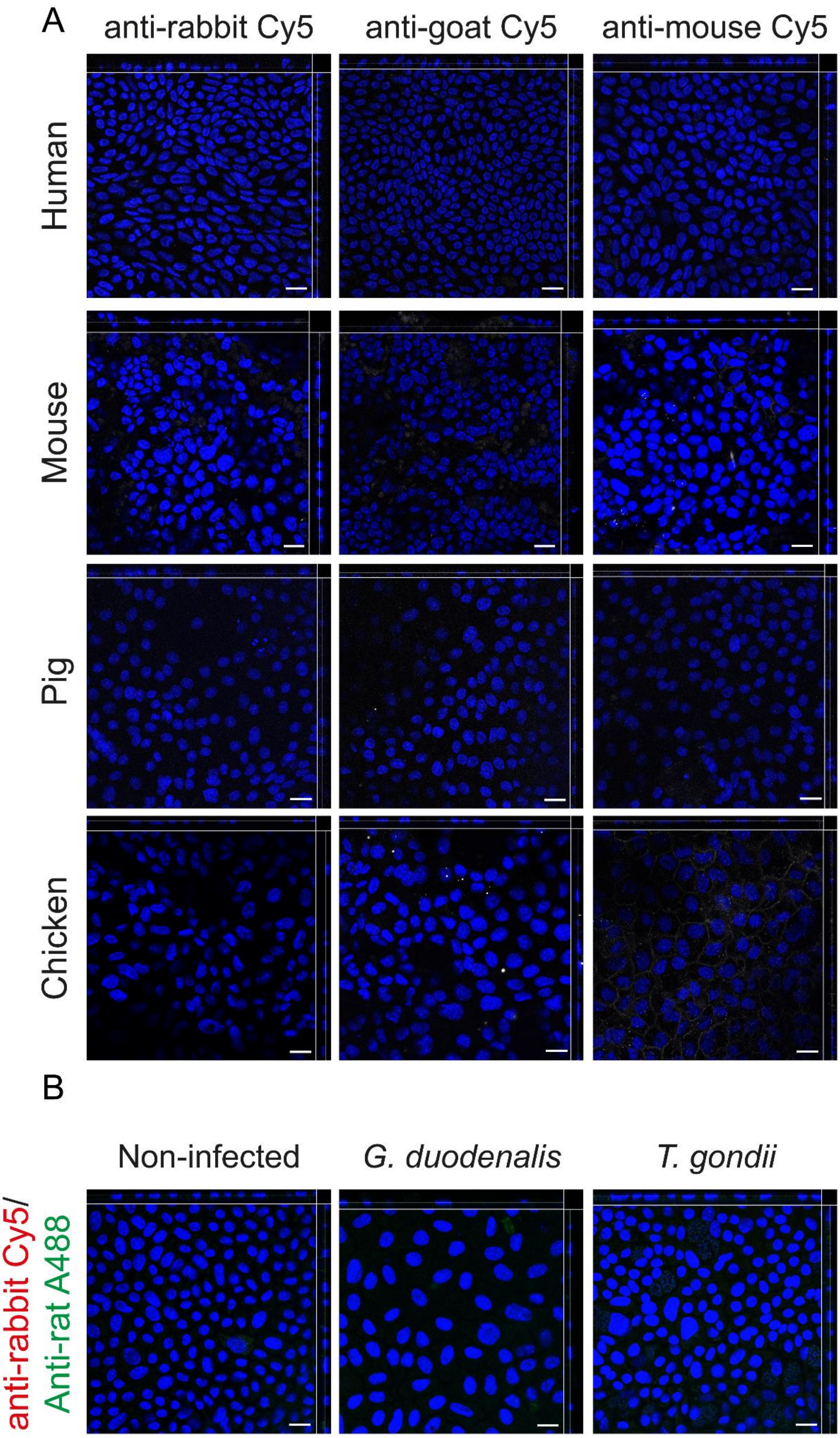

Supplementary Figure 5. Secondary antibody controls of (A) uninfected and (B) infected murine ODMs. No staining that would indicate cross-reactivity was detected. Scale bars indicate 20 µm

Alignment NHE3:

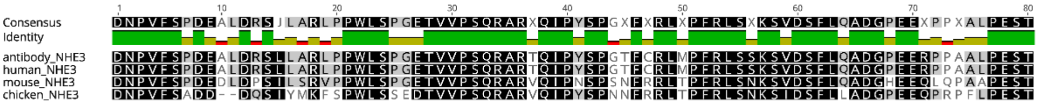

Alignment ACE2:

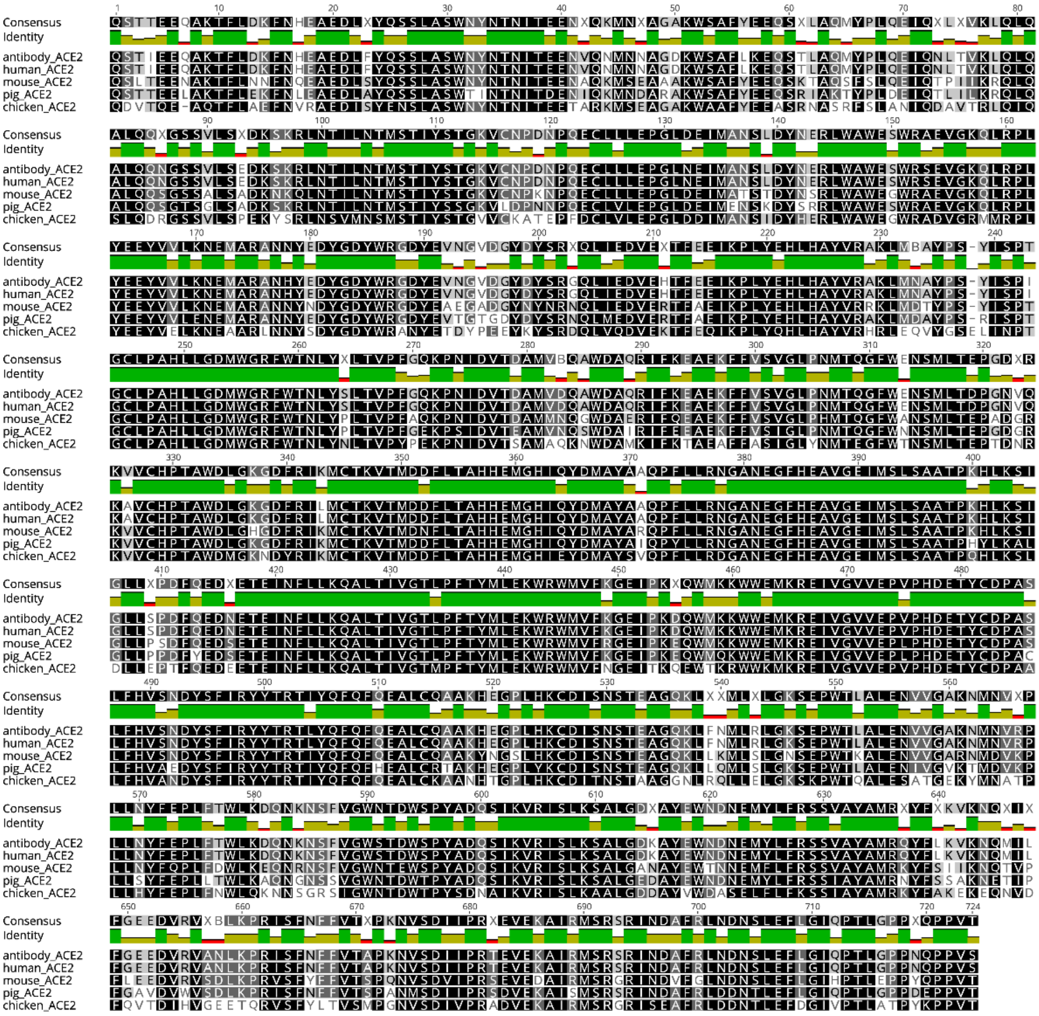

Alignment SOX9:

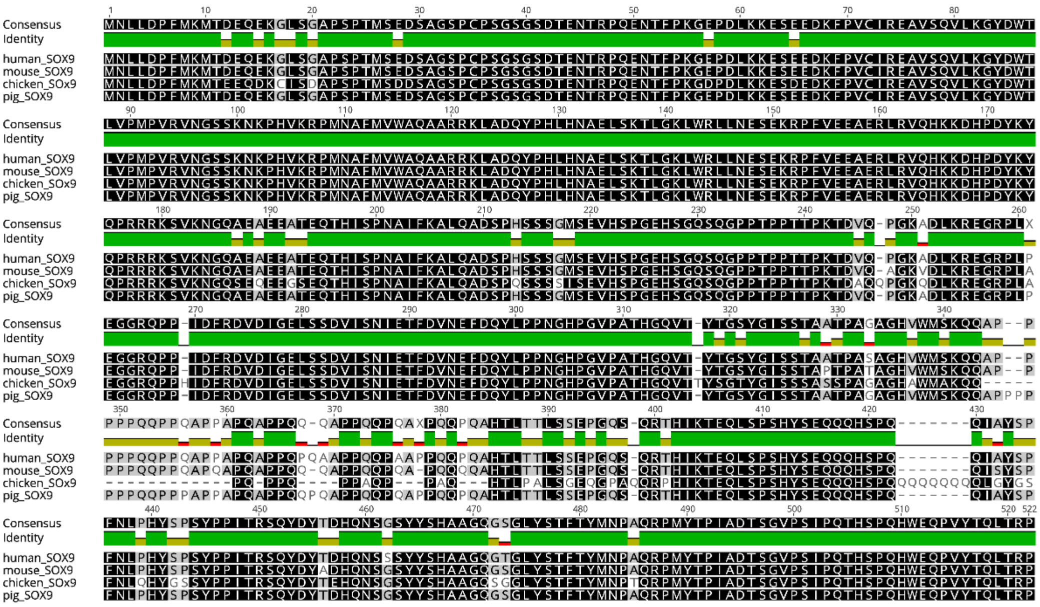

Figure continues on the next page

Alignment LYZ:

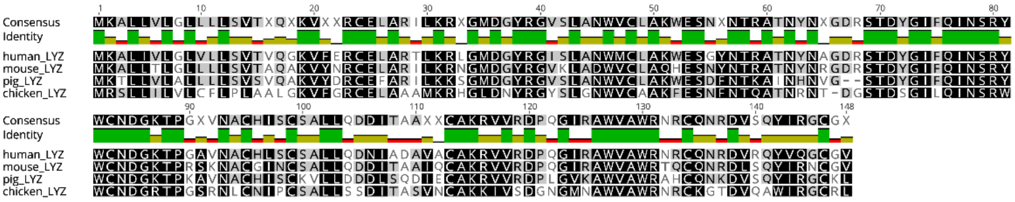

Alignment EZR:

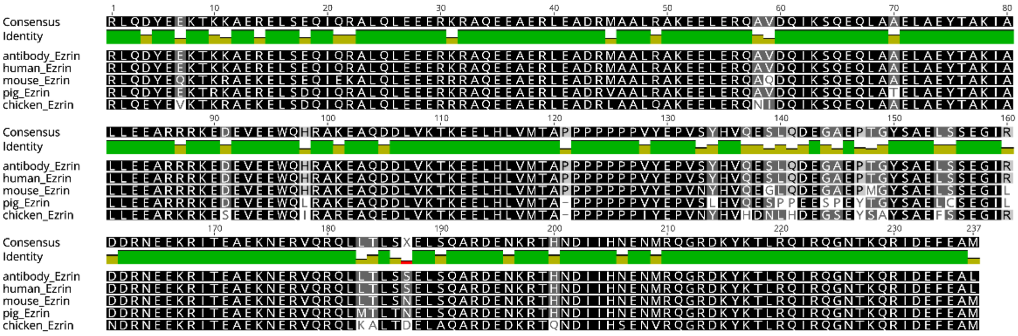

Alignment CHGA:

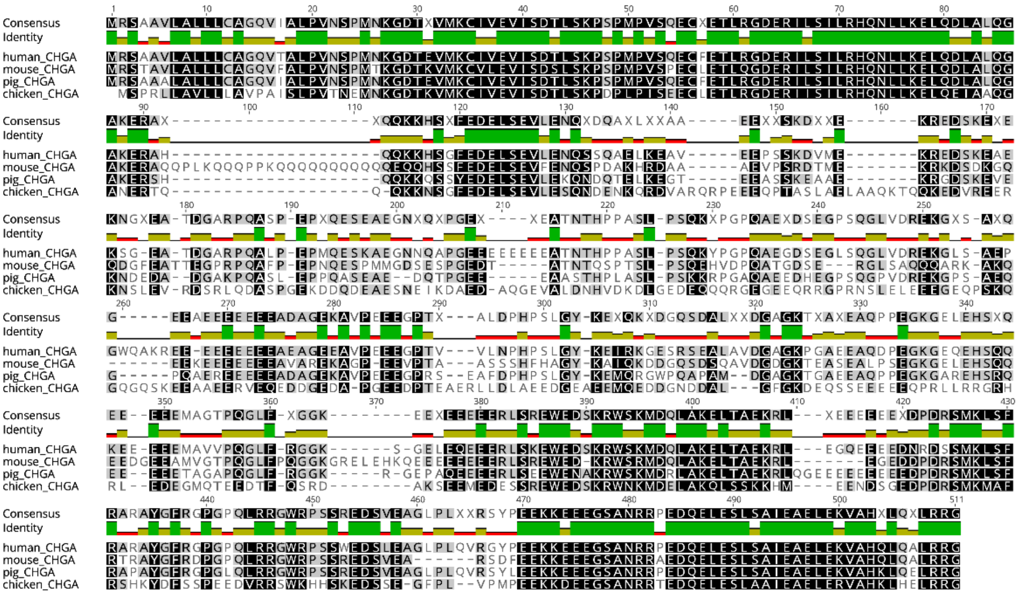

Alignment CDH1:

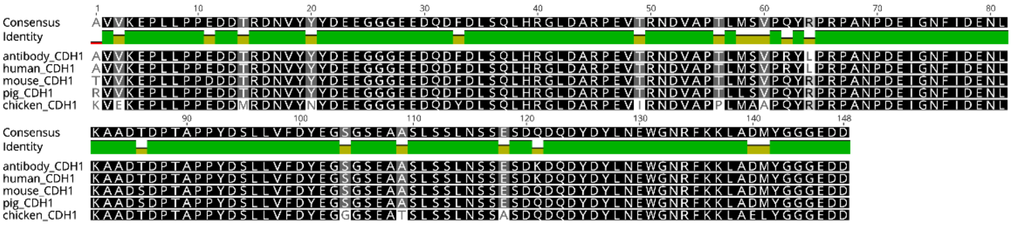

Supplementary Figure 6. Sequence similarities of the target epitopes from all four species recognized by the used antibodies. Alignments of protein sequences for all species were generated in Geneious Prime after being downloaded from the NCBI database. The available porcine protein sequence of NHE3 was only partially available and is therefore not included in the alignment. Thus, no conclusions about potential antibody binding could be derived and the respective IFA analysis was omitted in Figure 5. Epitope for the NHE3 antibody spans human sequence Asp756-Thr835, epitope for ACE2 spans human sequence Gln18-Ser740, epitope for EZR spans human sequence Arg350- Leu586 and epitope for CDH1 (ECAD) spans human sequence Ala735- Asp882. SOX9, LYZ and CHGA epitope sequences are not

specified in the respective datasheets and therefore complete alignments are shown. NCBI identifies were: hNHE3: NP_004165.2, mNHE3: NP_001074529.1, pNHE3: AAD24060.1, chNHE3: XP_004935132.1, hACE2: NP_001358344.1, mACE2: AAH26801.1, pACE2 NP_001116542.1, chACE2: QEQ50331.1, hSOX9: NP_000337.1, mSOX9: NP_035578.3, pSOX9: AHA92033.1, chSOX9: NP_989612.1, hLYZ: NP_000230.1, mLYZ: AAH61129.1, pLYZ: NP_999557.2, chLYZ: ACL81750.1, hEZR: NP_001104547.1, mEZR: NP_033536.2, pEZR: XP_013847913.2, chEZR: NP_990216.1, hCHGA: AAB53685.1, mCHGA: AAH26554.1, pCHGA: NP_001157477.2, chCHGA: XP_421330.1, hCDH1: NP_004351.1, mCDH1: AAH98501.1, pCDH1: NP_001156532.1, and chCDH1: NP_001034347.2.

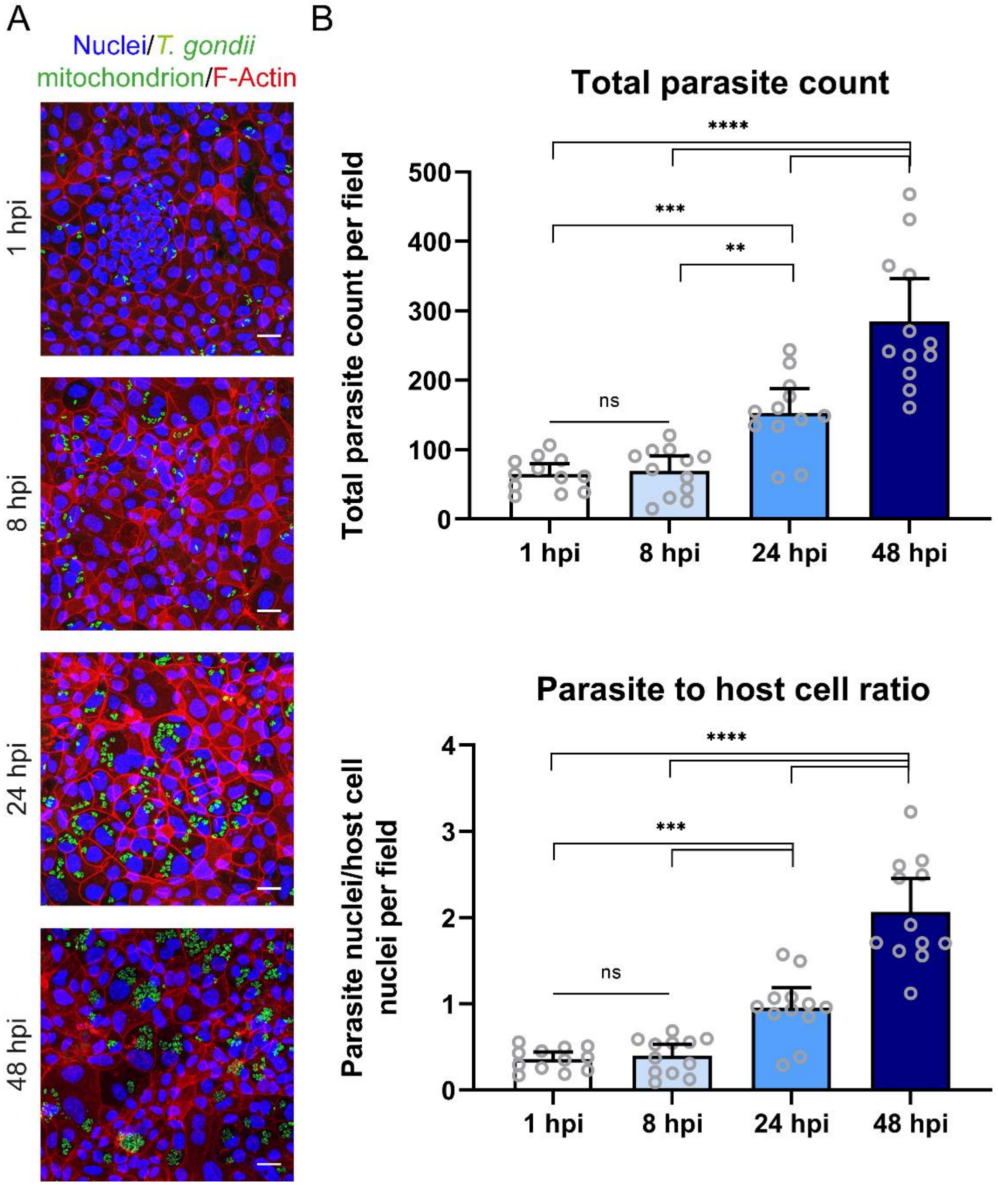

Supplementary Figure 7. Quantification of *T. gondii* parasite load over time in murine ODMs. (A) Representative confocal projections of infected murine ODMs with *T. gondii* (distinguished by its green tubular mitochondria) in TYI-S-33 medium. Scale bar indicates 20 µm. (B) Quantification of total parasite count per field and ratio of host and *T. gondii* nuclei per field (see Methods). Graphs show mean (± 95% CI) of a total of 12 measurements per timepoint of two independent experiments. Statistical significance was determined using a Two-Way ANOVA with Tukey's correction for multiple testing. ** $p < 0.01$, *** $p < 0.001$, **** $p < 0.0001$

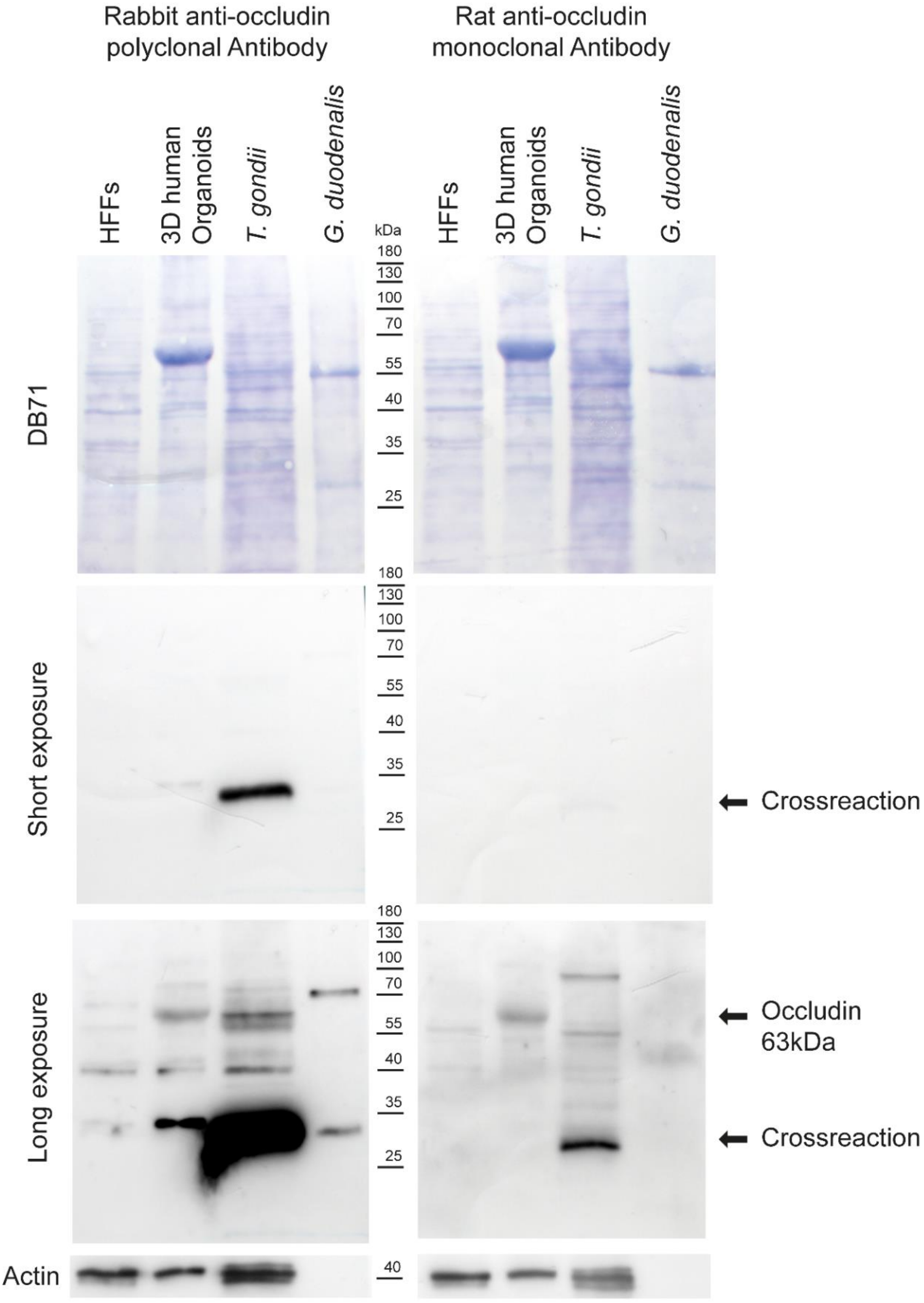

Supplementary Figure 8. Representative Western blots indicating strong cross-reactivity of antibodies against occludin with an unknown *T. gondii* protein. Bands in the *T. gondii* lanes can partly be explained by presence of left-over host cell proteins. Of note is that both antibodies, despite different clonality, show strong cross-reactivity to an unknown ~ 30 kDa *T. gondii* protein. Cross reactivity of the polyclonal antibody to *G. duodenalis* was also reproducible in IFAs. Note, the antibody for actin did not recognize the respective *G. duodenalis* protein for unknown reason; however, the DB71 staining confirmed presence of protein in the lysates. The experiment was repeated twice with similar results. Secondary antibody controls can be found in supplementary Figure 9.

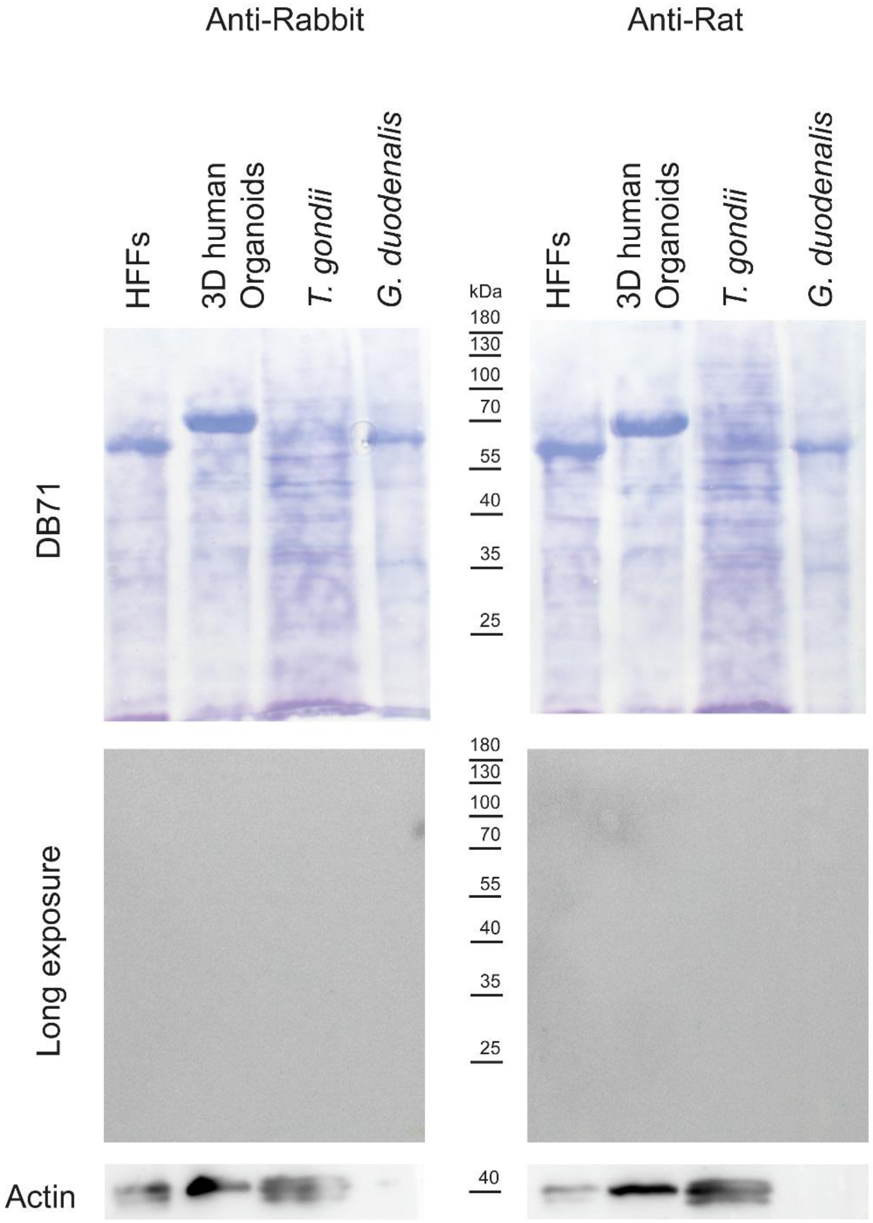

Supplementary Figure 9. Secondary antibody controls of HRP antibodies used in supplementary Figure 8. No bands were detected that would indicate cross-reactivity in any lane.

Supplementary references:

Bartfeld, S., Bayram, T., van de Wetering, M., Huch, M., Begthel, H., Kujala, P., et al. (2015). In Vitro Expansion of Human Gastric Epithelial Stem Cells and Their Responses to Bacterial Infection. *Gastroenterology* 148(1)**,** 126-136.e126. doi: 10.1053/j.gastro.2014.09.042.

Chen, J., Tellez, G., Richards, J.D., and Escobar, J. (2015). Identification of Potential Biomarkers for Gut Barrier Failure in Broiler Chickens. *Front Vet Sci* 2**,** 14. doi: 10.3389/fvets.2015.00014.

Forder, R.E., Nattrass, G.S., Geier, M.S., Hughes, R.J., and Hynd, P.I. (2012). Quantitative analyses of genes associated with mucin synthesis of broiler chickens with induced necrotic enteritis. *Poult Sci* 91(6)**,** 1335-1341. doi: 10.3382/ps.2011-02062.

Gal-Garber, O., Mabjeesh, S.J., Sklan, D., and Uni, Z. (2000). Partial sequence and expression of the gene for and activity of the sodium glucose transporter in the small intestine of fed, starved and refed chickens. *J Nutr* 130(9)**,** 2174-2179. doi: 10.1093/jn/130.9.2174.

Gonzalez, L.M., Williamson, I., Piedrahita, J.A., Blikslager, A.T., and Magness, S.T. (2013). Cell Lineage Identification and Stem Cell Culture in a Porcine Model for the Study of Intestinal Epithelial Regeneration. *PLOS ONE* 8(6)**,** e66465. doi: 10.1371/journal.pone.0066465.

Kim, K.A., Kakitani, M., Zhao, J., Oshima, T., Tang, T., Binnerts, M., et al. (2005). Mitogenic influence of human R-spondin1 on the intestinal epithelium. *Science* 309(5738)**,** 1256-1259. doi: 10.1126/science.1112521.

Kraft, M., Holthaus, D., Krug, S., Holland, G., Schulzke, J.-D., Aebischer, T., et al. (2020). Dissection of barrier dysfunction in organoid-derived human intestinal epithelia induced by Giardia duodenalis. *bioRxiv***,** 2020.2011.2017.384537. doi: 10.1101/2020.11.17.384537.

Li, J., Li, J., Jr., Zhang, S.Y., Li, R.X., Lin, X., Mi, Y.L., et al. (2018). Culture and characterization of chicken small intestinal crypts. *Poult Sci* 97(5)**,** 1536-1543. doi: 10.3382/ps/pey010.

Martorelli Di Genova, B., Wilson, S.K., Dubey, J.P., and Knoll, L.J. (2019). Intestinal delta-6-desaturase activity determines host range for Toxoplasma sexual reproduction. *PLoS Biol* 17(8)**,** e3000364. doi: 10.1371/journal.pbio.3000364.

Miyoshi, H., and Stappenbeck, T.S. (2013). In vitro expansion and genetic modification of gastrointestinal stem cells in spheroid culture. *Nat Protoc* 8(12)**,** 2471-2482. doi: 10.1038/nprot.2013.153.

Mulvaney, J.F., Amemiya, Y., Freeman, S.D., Ladher, R.K., and Dabdoub, A. (2015). Molecular cloning and functional characterisation of chicken Atonal homologue 1: a comparison with human Atoh1. *Biol Cell* 107(2)**,** 41-60. doi: 10.1111/boc.201400078.

Nakata, T., Ishiguro, M., Aduma, N., Izumi, H., and Kuroiwa, A. (2013). Chicken hemogen homolog is involved in the chicken-specific sex-determining mechanism. *Proc Natl Acad Sci U S A* 110(9)**,** 3417-3422. doi: 10.1073/pnas.1218714110.

Schneider, C.A., Rasband, W.S., and Eliceiri, K.W. (2012). NIH Image to ImageJ: 25 years of image analysis. *Nat Methods* 9(7)**,** 671-675. doi: 10.1038/nmeth.2089.

Thomsen-Zieger, N., Schachtner, J., and Seeber, F. (2003). Apicomplexan parasites contain a single lipoic acid synthase located in the plastid. *FEBS Lett* 547(1-3)**,** 80-86. doi: 10.1016/s0014-5793(03)00673-2.

VanDussen, K.L., Marinshaw, J.M., Shaikh, N., Miyoshi, H., Moon, C., Tarr, P.I., et al. (2015). Development of an enhanced human gastrointestinal epithelial culture system to facilitate patient-based assays. *Gut* 64(6)**,** 911-920. doi: 10.1136/gutjnl-2013-306651.

Wang, X., Spandidos, A., Wang, H., and Seed, B. (2012). PrimerBank: a PCR primer database for quantitative gene expression analysis, 2012 update. *Nucleic Acids Res* 40(Database issue)**,** D1144-1149. doi: 10.1093/nar/gkr1013.

Wang, Z., Yue, Y.X., Liu, Z.M., Yang, L.Y., Li, H., Li, Z.J., et al. (2019). Genome-Wide Analysis of the FABP Gene Family in Liver of Chicken (Gallus gallus): Identification,Dynamic Expression Profile, and RegulatoryMechanism. *Int J Mol Sci* 20(23). doi: 10.3390/ijms20235948.

Wells, J.M., Brummer, R.J., Derrien, M., MacDonald, T.T., Troost, F., Cani, P.D., et al. (2017). Homeostasis of the gut barrier and potential biomarkers. *Am J Physiol Gastrointest Liver Physiol* 312(3)**,** G171-g193. doi: 10.1152/ajpgi.00048.2015.

Yadgary, L., Yair, R., and Uni, Z. (2011). The chick embryo yolk sac membrane expresses nutrient transporter and digestive enzyme genes. *Poult Sci* 90(2)**,** 410-416. doi: 10.3382/ps.2010-01075.
